# Obesity attenuates gender differences in cardiovascular mortality

**DOI:** 10.1186/s12933-014-0144-5

**Published:** 2014-10-19

**Authors:** Xin Song, Adam G Tabák, Björn Zethelius, John S Yudkin, Stefan Söderberg, Tiina Laatikainen, Coen DA Stehouwer, Rachel Dankner, Pekka Jousilahti, Altan Onat, Peter M Nilsson, Ilhan Satman, Olga Vaccaro, Jaakko Tuomilehto, Qing Qiao, for the DECODE Study Group

**Affiliations:** Department of Public Health, Hjelt Institute, University of Helsinki, Helsinki, Finland; Department of Chronic Disease Prevention, National Institute for Health and Welfare, Helsinki, Finland; Department of Epidemiology and Public Health, University College London, London, UK; 1st Department of Medicine, Faculty of Medicine, Semmelweis University, Budapest, Hungary; Department of Public Health and Caring Sciences/Geriatrics, Uppsala University, Uppsala, Sweden; Medical Products Agency, Uppsala, Sweden; Department of Medicine, University College London, London, UK; Department of Public Health and Clinical Medicine, Umeå University, Umeå, Sweden; Baker IDI Heart and Diabetes Institute, Melbourne, Australia; Institute of Public Health and Clinical Nutrition, University of Eastern Finland, Kuopio, Finland; Hospital District of North Karelia, Joensuu, Finland; Department of Internal Medicine and Cardiovascular Research Institute Maastricht (CARIM), Maastricht University Medical Centre, Maastricht, The Netherlands; Unit for Cardiovascular Epidemiology, The Gertner Institute, Sheba Medical Center, Tel Hashomer, Israel; Division of Epidemiology and Prevention, School of Public Health, Sackler School of Medicine, Tel Aviv University, Tel Aviv, Israel; Department of Cardiology, Cerrahpaşa Medical Faculty, Istanbul, Turkey; Department of Clinical Sciences, Lund University, Malmö, Sweden; Center of Emergency Medicine, Skåne University Hospital, Malmö, Sweden; Istanbul University, Istanbul Medical Faculty, Istanbul, Turkey; Department of Clinical Medicine and Surgery, Federico II University, Naples, Italy; Center for Vascular Prevention, Danube University Krems, Krems, Austria; King Abdulaziz University, Jeddah, Saudi Arabia; Instituto de Investigacion Sanitaria del Hospital Universario LaPaz (IdiPAZ), Madrid, Spain; R&D AstraZeneca AB, Mölndal, Sweden

**Keywords:** Obesity, Gender, Cardiovascular disease mortality

## Abstract

**Background:**

To estimate cardiovascular disease (CVD) mortality in relation to obesity and gender.

**Methods:**

Data from 11 prospective cohorts from four European countries including 23 629 men and 21 965 women, aged 24 to 99 years, with a median follow-up of 7.9 years were analyzed. Hazards ratios (HR) for CVD mortality in relation to baseline body mass index (BMI), waist circumference (WC), waist-to-hip ratio (WHR) and waist-to-height ratio (WHtR) were estimated using Cox proportional hazards models with age as the timescale.

**Results:**

Men had higher CVD mortality than women in all four BMI categories (<25.0, 25.0-29.9, 30.0-34.9 and ≥35.0 kg/m^2^). Compared with the lowest BMI category in women, multivariable adjusted HRs (95% confidence intervals) for higher BMI categories are 1.0 (0.8-1.4), 1.6 (1.1-2.1) and 2.8 (2.0-3.8) in women and 2.8 (2.2-3.6), 3.1 (2.5-3.9), 3.8 (2.9-4.9) and 5.4 (3.8-7.7) in men, respectively. Similar findings were observed for abdominal obesity defined by WC, WHR or WHtR. The gender difference was slightly smaller in obese than in non-obese individuals; but the interaction was statistically significant only between gender and WC (p = 0.02), and WHtR (p = 0.01). None of the interaction terms was significant among non-diabetic individuals.

**Conclusions:**

Men had higher CVD mortality than women across categories of anthropometric measures of obesity. The gender difference was attenuated in obese individuals, which warrants further investigation.

**Electronic supplementary material:**

The online version of this article (doi:10.1186/s12933-014-0144-5) contains supplementary material, which is available to authorized users.

## Background

Cardiovascular diseases (CVD) are still the leading cause of mortality in both men and women [1]. Further, women are known to have a much lower risk for CVD mortality than men. There is substantial evidence of a gender difference in cardiac autonomic modulation [2-5], lipid and glucose metabolism [6-9], sex hormones [4,10-14] and cytokines [15-19]. On average, women would have augmented sympathetic inhibition, higher cardiac vagal tone, higher heart rate variability, lower susceptibility to arrhythmias, and decreased myocardial contractility than men [2,3,20], leading to a preponderance of vagal over sympathetic control of cardiac function [2-5]. Moreover, before menopause, women generally have lower levels of serum total and low-density lipoprotein cholesterol (TC and LDL-C), triglycerides and apolipoprotein B and higher levels of high-density lipoprotein cholesterol (HDL-C) and apolipoprotein A-I than their male counterparts [6,21-23] although TC and LDL-C increase in women after menopause [21,22]. However, this female advantage is abrogated with diabetes and aging [24-32], perhaps as a consequence of diabetes inducing higher levels of inflammatory mar-kers and higher rates of nitric oxide release in women compared with men, resulting in reduced protective effects of estrogen on body fat distribution and insulin action, or a more impaired endothelial function in women than in men [10,33]. The prevalence of obesity is rising worldwide, leading to an increased risk for diabetes and CVD [34,35]. It is not clear, however, whether the gender difference in CVD mortality remains after accounting for the increased development of obesity.

The aim of this study was to assess the risk of CVD mortality in relation to obesity and gender [1] in the general population and [2] separately for those with or without diabetes at baseline.

## Subjects and methods

### Study population

This analysis was based on 11 cohorts from the DECODE (Diabetes Epidemiology: Collaborative analysis Of Diagnostic criteria in Europe) study including 45 594 individuals (48.2% women) aged 24–99 at baseline, from four European countries (Finland, Sweden, Turkey and UK). Information on weight, height, waist and hip circumferences, self-reported smoking status, leisure-time physical activity, and history of diabetes mellitus were available. In addition, 20 270 individuals (44.7% women) had data on fasting or 2-h plasma glucose (FPG and 2hPG) from a 75 g oral glucose tolerance test. Individual participant data from each cohort were sent to the National Institute for Health and Welfare in Helsinki, Finland for collaborative data analyses. Studies included were approved by local ethics committees, and the analysis plan was approved by the ethics committee of the National Institute for Health and Welfare, Helsinki, Finland.

### Definition of covariates

Height and weight were measured without shoes and with light clothing. Waist circumference (WC) was measured midway between the lower rib margin and iliac crest. Hip circumference was measured at the level of the widest circumference over the greater trochanters. Body mass index (BMI) was calculated as weight in kilograms divided by the square of height in meters. Waist-to-hip ratio (WHR) and waist-to-height ratio (WHtR) were calculated as waist circumference divided by hip circumference or height, respectively. Participants were classified into five categories: underweight (BMI <18.5 kg/m^2^), normal weight (BMI 18.5-24.9 kg/m^2^), overweight (BMI 25.0-29.9 kg/m^2^), obesity (BMI 30.0-34.9 kg/m^2^) and severe obesity (BMI ≥35.0 kg/m^2^), according to the WHO classification criteria [36]. Due to the low number of participants in the underweight category in this study population, the underweight and normal weight groups were combined for analysis. Abdominal obesity was defined as the sex-specific top quartile of WC, WHR or WHtR (WC ≥99 cm, WHR ≥0.97, and WHtR ≥0.57 for males; WC ≥90 cm, WHR ≥0.85, and WHtR ≥0.56 for females, respectively). Participants reporting reading, watching TV, housework, sewing and walking <1 km daily were defined as physically inactive; all those engaging in higher levels of physical activity were defined as physically active. Based on responses to the questionnaire, smoking status at baseline was classified into three categories of never, former and current smokers. Diabetes was defined as either a history of diabetes at baseline or an FPG level ≥7.0 mmol/L and/or a 2hPG level ≥11.1 mmol/L [37].

### Definition of fatal events

Vital status and causes of death were recorded for all participants. CVD mortality was defined according to the International Classification of Disease codes 401–448 (9th revision) or I10–I79 (10th revision).

### Statistical analyses

The absolute CVD mortality rate per 10 000 person-years of follow-up for each obesity and gender category was calculated. Cox proportional hazards models were used to estimate hazard ratios (HR) and 95% confidence intervals (CIs) for CVD mortality, adjusting for baseline smoking status, leisure-time physical activity and cohort, using attained age as the timescale and non-obese women as the reference group. To investigate whether the gender difference in CVD mortality was different in non-obese from obese participants, we checked for the interaction between sex and obesity in the Cox models, using a chi-squared log-likelihood ratio test. STATA version 11 (StataCorp, College Station, TX, USA) was used.

## Results

Table 1 provides baseline characteristics of the cohorts. Median follow-up time varied between 2.5 and 21.8 years in the different cohorts. Lean females were younger, more abdominally obese and had low prevalence of diabetes, while obese females were older, less abdominally obese and had higher prevalence of diabetes at baseline, compared with their respective male counterparts (Table 2). More men than women were smokers and physically active. Men tended to have higher mean values of systolic blood pressure and fasting plasma glucose, and a worse lipid profile than women, regardless of BMI categories. Similar characteristics were observed among non-diabetic individuals (Additional file 1: Table S1). But diabetic men tended to be older and less physically active. Similar gender specific baseline characteristics were observed within the categories of abdominal obesity defined by sex-specific quartiles of anthropometric measures of WC, WHR and WHtR (Additional file 1: Table S2).

**Table 1:** **Baseline characteristics and follow-up information of the survey participants**

**Study**	**Number of participants**	**Age (years)**	**BMI (kg/m** ^**2**^ **)**	**WC (cm)**	**WHR**	**WHtR**	**Median follow-up (years)**	**No. (%) of CVD deaths**
**Men**								
Finland								
FINRISK (1987)	2541	43.8 (11.2)	26.7 (3.7)	92.2 (10.7)	0.90 (0.06)	0.53 (0.06)	21.8	293 (11.5)
FINRISK (1992)	2570	44.3 (11.2)	26.6 (3.9)	93.9 (11.2)	0.92 (0.07)	0.53 (0.07)	16.8	152 (5.9)
FINRISK (1997)	3788	48.6 (13.5)	26.9 (3.9)	94.5 (11.3)	0.93 (0.07)	0.54 (0.07)	11.8	205 (5.4)
FINRISK (2002)	3808	48.0 (13.0)	27.2 (4.1)	95.4 (11.8)	0.97 (0.07)	0.54 (0.07)	6.8	69 (1.8)
Sweden								
Northern Sweden MONICA (1986)	671	46.0 (11.4)	25.4 (3.4)	92.4 (9.2)	0.94 (0.05)	0.52 (0.06)	20.5	49 (7.3)
Northern Sweden MONICA (1990)	761	45.0 (11.3)	25.8 (3.3)	91.4 (9.1)	0.93 (0.06)	0.52 (0.05)	16.5	30 (3.9)
Northern Sweden MONICA (1994)	877	49.6 (14.0)	26.2 (3.7)	93.4 (10.1)	0.94 (0.06)	0.53 (0.06)	12.5	37 (4.2)
Northern Sweden MONICA (1999)	869	50.6 (14.3)	26.7 (3.5)	95.3 (9.7)	0.92 (0.07)	0.54 (0.06)	7.4	14 (1.6)
Northern Sweden MONICA (2004)	864	50.9 (14.1)	27.2 (3.9)	96.4 (10.7)	0.96 (0.06)	0.54 (0.06)	2.5	0
Turkey								
TARFS (1998–2002)	1580	53.2 (12.4)	26.4 (4.0)	94.3 (11.0)	0.93 (0.07)	0.56 (0.06)	7.9	55 (3.5)
UK								
Whitehall II (1991–1993)	5300	49.3 (6.0)	25.1 (3.2)	87.4 (9.2)	0.90 (0.06)	0.50 (0.05)	5.9	41 (0.8)
Total	23 629	48.0 (11.8)	26.4 (3.8)	92.6 (11.0)	0.93 (0.07)	0.53 (0.07)	7.9	945 (4.0)
**Women**								
Finland								
FINRISK (1987)	2812	43.7 (11.4)	26.0 (4.9)	79.4 (11.2)	0.78 (0.06)	0.49 (0.07)	21.8	119 (4.2)
FINRISK (1992)	2828	44.0 (11.5)	25.7 (4.9)	80.2 (11.7)	0.79 (0.07)	0.49 (0.08)	16.9	55 (1.9)
FINRISK (1997)	3788	46.1 (12.7)	26.1 (4.9)	81.3 (12.2)	0.80 (0.07)	0.50 (0.08)	11.8	61 (1.6)
FINRISK (2002)	4383	46.6 (13.0)	26.4 (5.0)	83.6 (12.6)	0.84 (0.06)	0.52 (0.08)	6.8	14 (0.3)
Sweden								
Northern Sweden MONICA (1986)	685	45.6 (11.1)	25.0 (4.4)	85.3 (12.2)	0.86 (0.07)	0.52 (0.08)	20.5	18 (2.6)
Northern Sweden MONICA (1990)	793	44.8 (11.4)	25.0 (4.4)	79.4 (11.0)	0.81 (0.06)	0.49 (0.07)	16.5	12 (1.5)
Northern Sweden MONICA (1994)	902	49.4 (14.0)	25.8 (4.7)	84.2 (12.4)	0.83 (0.08)	0.52 (0.08)	12.5	16 (1.8)
Northern Sweden MONICA (1999)	900	50.1 (14.1)	26.3 (4.6)	84.9 (11.8)	0.82 (0.07)	0.52 (0.08)	7.5	3 (0.3)
Northern Sweden MONICA (2004)	909	49.7 (13.9)	26.6 (5.1)	86.6 (12.9)	0.85 (0.07)	0.53 (0.08)	2.5	0
Turkey								
TARFS (1998–2002)	1619	52.7 (12.3)	28.8 (5.6)	90.7 (12.7)	0.84 (0.08)	0.58 (0.09)	7.9	35 (2.2)
UK								
Whitehall II (1991–1993)	2346	50.2 (6.1)	25.7 (4.7)	75.5 (11.7)	0.77 (0.07)	0.47 (0.07)	5.8	6 (0.3)
Total	21 965	46.9 (12.3)	26.2 (5.0)	82.0 (12.6)	0.81 (0.07)	0.51 (0.08)	11.8	339 (1.5)

*Abbreviations*: *BMI* body mass index, *WC* waist circumference, *WHR* waist-to-hip ratio, *WHtR* waist-to-height ratio, *CVD* cardiovascular disease.

Data are means (standard deviations) or as noted.

**Table 2:** **Baseline characteristics of participants by body mass index and sex**

	**<25.0 kg/m** ^**2**^			**25.0-29.9 kg/m** ^**2**^			**30.0-34.9 kg/m** ^**2**^			**≥35.0 kg/m** ^**2**^		
	**M (n = 9275)**	**F (n = 10 519)**	P*	**M (n = 10 738)**	**F (n = 7142)**	P*	**M (n = 3017)**	**F (n = 2962)**	P*	**M (n = 599)**	**F (n = 1342)**	P*
Age (years)	45.6 (11.8)	42.9 (11.7)	0.00	49.0 (11.5)	49.7 (11.7)	0.00	51.3 (11.3)	52.3 (11.5)	0.00	50.6 (11.3)	52.4 (11.1)	0.00
Abdominal obesity (%)^†^												
WC	0.6	1.3	0.00	26.9	24.2	0.00	91.4	79.3	0.00	99.7	98.1	0.01
WHR	5.3	8.6	0.00	27.4	30.1	0.00	64.3	52.6	0.00	85.1	66.0	0.00
WHtR	0.6	1.2	0.00	23.7	23.1	0.35	89.5	81.0	0.00	99.7	99.0	0.17
Diabetes status (%)^‡^			0.27			0.00			0.00			0.04
Newly diagnosed diabetes	11.3	10.2		13.4	16.0		19.5	24.8		24.4	32.6	
Known diabetes	3.2	3.2		6.0	5.4		11.6	10.2		17.2	15.0	
Smoking status (%)			0.00			0.00			0.00			0.00
Current smokers	25.6	22.1		23.7	17.6		25.3	15.1		25.2	13.5	
Former smokers	27.9	17.3		34.6	18.7		38.7	18.7		38.2	18.7	
Physically active (%)	83.1	78.6	0.00	80.4	77.7	0.00	73.3	70.7	0.03	61.9	62.1	0.96
SBP (mmHg)^§^	127 (17)	124 (18)	0.00	135 (18)	133 (21)	0.00	142 (19)	140 (21)	0.00	147 (20)	145 (22)	0.04
FPG (mmol/L)^§^	5.3 (0.9)	5.2 (0.8)	0.00	5.5 (1.1)	5.4 (1.0)	0.00	5.9 (1.5)	5.7 (1.3)	0.00	6.4 (2.2)	5.9 (1.7)	0.00
TG (mmol/L)^§^	1.3 (0.9)	1.0 (0.6)	0.00	1.8 (1.2)	1.4 (0.8)	0.00	2.2 (1.5)	1.6 (1.0)	0.00	2.5 (1.9)	1.9 (1.2)	0.00
HDL-C (mmol/L)^§^	1.4 (0.4)	1.7 (0.4)	0.00	1.2 (0.3)	1.5 (0.4)	0.00	1.1 (0.3)	1.4 (0.4)	0.00	1.1 (0.3)	1.3 (0.3)	0.00
Total-C (mmol/L)^§^	5.7 (1.2)	5.5 (1.2)	0.00	6.0 (1.2)	5.9 (1.3)	0.00	6.0 (1.2)	5.9 (1.2)	0.04	5.9 (1.2)	5.8 (1.2)	0.61

*Abbreviations*: *M* male, *F* female, *WC* waist circumference, *WHR* waist-to-hip ratio, *WHtR* waist-to-height ratio, *SBP* systolic blood pressure, *FPG* fasting plasma glucose, *TG* triglyceride, *HDL-C* high-density lipoprotein cholesterol, *Total-C* total cholesterol.

Data are means (standard deviations) or as noted.

*Difference between men and women.

^†^The top quartile of WC, WHR or WHtR in women (90 cm, 0.85 or 0.56) and in men (99 cm, 0.97 or 0.57) was used as abdominal obesity group.

^‡^21 555 individuals with information on diabetes status, including 1285 with known diagnosis of diabetes and 20 270 with available measurement of fasting plasma glucose and/or 75 g 2-h oral glucose tolerance test (44.7% women).

^§^45 573 individuals with available measurement of SBP, 20 759 for FPG, 36 834 for TG, 41 808 for HDL-C and 45 353 for Total-C.

During the median follow-up of 7.9 years, 945 (4.0%) men and 339 (1.5%) women died from CVD. Absolute rates and age- or multivariate-adjusted HRs for CVD mortality are shown across BMI categories or sex-specific quartiles of anthropometric measures of abdominal obesity (Table 3). Males had higher CVD mortality rates and higher hazard ratios across BMI categories, and categories of abdominal obesity than females, but the sex difference tended to be attenuated in the obese categories. The interaction was statistically significant between gender and WC (p = 0.02), and WHtR (p = 0.01), but not significant with BMI and WHR (Table 3). For most studies, study-specific HRs were within 10% of the pooled estimate, although there was heterogeneity by some studies for the HRs of sex-ratio within BMI 25.0-29.9 kg/m^2^ (I^2^ = 95.5%, P <0.05, Additional file 1: Table S3). However, exclusion of any study in the analysis had little overall influence on the main results (Additional file 1: Table S4). The finding persisted when the analysis was restricted to individuals without baseline diabetes, but the gender-obesity interaction was no longer significant in the non-diabetic population (Table 4). In addition, the gender difference among diabetic individuals diminished especially for non-obese ones.

**Table 3:** **Mortality rate per 10 000 person-years and multivariate-adjusted hazard ratios for cardiovascular disease mortality in men and women by body mass index categories or sex-specific quartiles of anthropometric measures of abdominal obesity**

	**BMI, kg/m** ^**2**^				WC, cm*		WHR*		WHtR*	
	**<25.0**	**25.0-29.9**	**30.0-34.9**	**≥35.0**	**<90 (F)/99 (M)**	**≥90 (F)/99 (M)**	**<0.85 (F)/0.97 (M)**	**≥0.85 (F)/0.97 (M)**	**<0.56 (F)/0.57 (M)**	**≥0.56 (F)/0.57 (M)**
No. of CVD deaths										
Women	90	107	81	61	173	166	195	144	159	180
Men	241	459	198	47	506	439	555	390	480	465
Mortality rate (95% CI)										
Women	7 (6-9)	13 (11-16)	25 (20-31)	45 (34-58)	9 (8-10)	29 (25-34)	10 (9-11)	27 (22–31)	8 (7–10)	32 (27–37)
Men	25 (22-29)	41 (37-45)	63 (54-72)	78 (57-104)	28 (26-31)	67 (61-74)	29 (27-32)	70 (63–77)	26 (24–28)	77 (70–84)
Sex ratio in CVD mortality rate (M/F)	3.5	3.0	2.5	1.7	3.2	2.3	3.0	2.6	3.2	2.4
Hazard ratios (95% CI)^†^										
Women	1.0 (Reference)	1.0 (0.8-1.3)	1.5 (1.1-2.0)	2.8 (2.1-3.9)	1.0 (Reference)	1.9 (1.5-2.3)	1.0 (Reference)	1.6 (1.3-2.0)	1.0 (Reference)	2.0 (1.6-2.5)
Men	3.1 (2.5-4.0)	3.5 (2.8-4.3)	4.5 (3.5-5.7)	6.5 (4.6-9.3)	3.2 (2.7-3.8)	4.8 (4.0-5.7)	3.0 (2.6-3.6)	4.5 (3.8-5.3)	3.2 (2.7-3.9)	5.1 (4.2-6.1)
Sex ratio in CVD mortality risk (M/F)	3.1 (2.5-4.0)	3.5 (2.8-4.3)	3.0 (2.3-3.8)	2.3 (1.6-3.4)	3.2 (2.7-3.8)	2.6 (2.1-3.1)	3.0 (2.6-3.6)	2.7 (2.3-3.3)	3.2 (2.7-3.9)	2.6 (2.2-3.0)
Hazard ratios (95% CI)^‡^										
Women	1.0 (Reference)	1.0 (0.8-1.4)	1.6 (1.1-2.1)	2.8 (2.0-3.8)	1.0 (Reference)	1.9 (1.6-2.4)	1.0 (Reference)	1.8 (1.5-2.3)	1.0 (Reference)	2.1 (1.7-2.6)
Men	2.8 (2.2-3.6)	3.1 (2.5-3.9)	3.8 (2.9-4.9)	5.4 (3.8-7.7)	2.9 (2.4-3.4)	4.1 (3.4-5.0)	2.7 (2.3-3.2)	4.2 (3.5-5.1)	3.0 (2.5-3.6)	4.4 (3.6-5.3)
Sex ratio in CVD mortality risk (M/F)	2.8 (2.2-3.6)	3.0 (2.4-3.7)	2.4 (1.8-3.2)	2.0 (1.3-2.9)	2.9 (2.4-3.4)	2.1 (1.8-2.6)§	2.7 (2.3-3.2)	2.3 (1.9-2.8)	3.0 (2.5-3.6)	2.1 (1.8-2.5)§

*Abbreviations*: *BMI* body mass index, *WC* waist circumference, *WHR* waist-to-hip ratio, *WHtR* waist-to-height ratio, *F* female, *M* male, *CVD* cardiovascular disease, *CI* confidence intervals.

*The top quartile of WC, WHR or WHtR in women (90 cm, 0.85 or 0.56) and in men (99 cm, 0.97 or 0.57) was used as the abdominal obesity group.

^†^Hazard ratios (95% CI) from the Cox proportional hazards model using attained age as time-scale.

^‡^Hazard ratios (95% CI) from the Cox proportional hazards model adjusted for baseline smoking status, leisure-time physical activity, and cohort using attained age as time-scale.

^§^Significance level = 0.05 for interactions between obesity and gender.

**Table 4:** **Mortality rate per 10 000 person-years and multivariate adjusted hazard ratios for cardiovascular disease mortality among non-diabetic and diabetic individuals by sex and body mass index categories or sex-specific quartiles of anthropometric measures of abdominal obesity**

	**BMI, kg/m** ^**2**^				WC, cm*		WHR*		WHtR*	
	**<25.0**	**25.0-29.9**	**30.0-34.9**	**≥35.0**	**<90 (F)/99 (M)**	**≥90 (F)/99 (M)**	**<0.85 (F)/0.97 (M)**	**≥0.85 (F)/0.97 (M)**	**<0.56 (F)/0.57 (M)**	**≥0.56 (F)/0.57 (M)**
Non-diabetic (n = 17 056)										
Mortality rate (95% CI)										
Women	9 (6-13)	14 (10-19)	29 (20-41)	50 (30-76)	12 (9-15)	29 (22-39)	14 (11-17)	24 (17-32)	11 (9-14)	32 (24-41)
Men	26 (21-32)	41 (35-48)	57 (44-73)	45 (18-92)	30 (26-34)	59 (50-71)	31 (27-35)	60 (49-71)	28 (24-33)	66 (56-78)
Sex ratio in CVD mortality rate (M/F)	2.9	3.0	2.0	0.9	2.5	2.0	2.3	2.5	2.6	2.1
Hazard ratios (95% CI)^†^										
Women	1.0 (Reference)	1.0 (0.7-1.6)	1.8 (1.1-3.0)	3.3 (1.9-5.8)	1.0 (Reference)	1.8 (1.3-2.6)	1.0 (Reference)	1.5 (1.0-2.2)	1.0 (Reference)	2.0 (1.4-2.8)
Men	3.6 (2.4-5.4)	3.9 (2.7-5.7)	4.6 (3.0-7.0)	4.1 (1.8-9.4)	3.3 (2.5-4.3)	4.4 (3.3-5.8)	3.0 (2.3-3.8)	4.1 (3.1-5.4)	3.3 (2.5-4.4)	4.7 (3.5-6.3)
Sex ratio in CVD mortality risk (M/F)	3.6 (2.4-5.4)	3.7 (2.7-5.3)	2.5 (1.6-3.8)	1.3 (0.5-3.0)	3.3 (2.5-4.3)	2.4 (1.7-3.3)	3.0 (2.3-3.8)	2.7 (1.9-3.9)	3.3 (2.5-4.4)	2.4 (1.8-3.3)
Hazard ratios (95% CI)^‡^										
Women	1.0 (Reference)	1.1 (0.7-1.7)	1.9 (1.2-3.0)	3.3 (1.9-5.8)	1.0 (Reference)	1.9 (1.4-2.8)	1.0 (Reference)	1.8 (1.2-2.6)	1.0 (Reference)	2.0 (1.4-2.9)
Men	3.2 (2.1-4.8)	3.4 (2.3-4.9)	3.9 (2.5-6.0)	3.8 (1.7-8.6)	2.9 (2.2-3.8)	3.8 (2.8-5.1)	2.6 (2.0-3.3)	4.1 (3.1-5.6)	2.9 (2.2-3.9)	4.0 (2.9-5.4)
Sex ratio in CVD mortality risk (M/F)	3.2 (2.1-4.8)	3.1 (2.2-4.4)	2.1 (1.4-3.2)	1.2 (0.5-2.7)	2.9 (2.2-3.8)	2.0 (1.4-2.7)	2.6 (2.0-3.3)	2.3 (1.6-3.3)	2.9 (2.2-3.9)	2.0 (1.4-2.7)
**Diabetic (n = 4499)**										
Mortality rate (95% CI)										
Women	39 (22–63)	52 (36–74)	57 (37–84)	70 (43–407)	40 (28–57)	64 (49–83)	37 (26–51)	73 (55–94)	35 (23–51)	66 (51–83)
Men	54 (36–77)	118 (95–144)	138 (103–182)	146 (80–245)	80 (64–99)	138 (113–167)	89 (72–108)	130 (105–160)	84 (67–105)	128 (105–154)
Sex ratio in CVD mortality rate (M/F)	1.4	2.3	2.4	2.1	2.0	2.1	2.4	1.8	2.4	1.9
Hazard ratios (95% CI)^†^										
Women	1.0 (Reference)	1.0 (0.6-1.9)	1.0 (0.5-1.8)	1.3 (0.7-2.5)	1.0 (Reference)	1.1 (0.7-1.7)	1.0 (Reference)	1.3 (0.8-1.9)	1.0 (Reference)	1.2 (0.8-1.9)
Men	1.3 (0.7-2.3)	2.2 (1.3-3.8)	2.5 (1.4-4.4)	2.7 (1.3-5.7)	1.8 (1.2-2.7)	2.4 (1.6-3.5)	2.2 (1.5-3.2)	2.4 (1.6-3.6)	2.2 (1.4-3.4)	2.3 (1.5-3.6)
Sex ratio in CVD mortality risk (M/F)	1.3 (0.7-2.3)	2.2 (1.5-3.3)	2.6 (1.6-4.1)	2.1 (1.1-4.2)	1.8 (1.2-2.7)	2.2 (1.6-3.0)	2.2 (1.5-3.2)	1.9 (1.4-2.7)	2.2 (1.4-3.4)	1.9 (1.4-2.6)
Hazard ratios (95% CI)^‡^										
Women	1.0 (Reference)	1.0 (0.5-1.8)	0.9 (0.5-1.7)	1.0 (0.5-1.9)	1.0 (Reference)	1.2 (0.8-1.8)	1.0 (Reference)	1.3 (0.9-2.0)	1.0 (Reference)	1.3 (0.8-2.1)
Men	1.3 (0.7-2.4)	1.9 (1.1-3.2)	1.9 (1.1-3.5)	1.6 (0.8-3.4)	1.9 (1.2-2.9)	2.1 (1.4-3.2)	2.1 (1.4-3.2)	2.3 (1.5-3.5)	2.2 (1.4-3.6)	2.3 (1.4-3.6)
Sex ratio in CVD mortality risk (M/F)	1.3 (0.7-2.4)	1.9 (1.3-2.9)	2.2 (1.3-3.6)	1.7 (0.9-3.4)	1.9 (1.2-2.9)	1.8 (1.3-2.6)	2.1 (1.4-3.2)	1.7 (1.2-2.4)	2.2 (1.4-3.6)	1.7 (1.2-2.4)

*Abbreviations*: *BMI* body mass index, *WC* waist circumference, *WHR* waist-to-hip ratio, *WHtR* waist-to-height ratio, *F* female, *M* male, *CI* confidence intervals.

*The top quartile of WC, WHR or WHtR in women (90 cm, 0.85 or 0.56) and in men (99 cm, 0.97 or 0.57) was used as the obesity group.

^†^Hazard ratios (95% CI) from the Cox proportional hazards model using attained age as time-scale. ^‡^Hazard ratios (95% CI) from the Cox proportional hazards model adjusted for baseline smoking status, leisure-time physical activity, and cohort using attained age as time-scale.

The findings for BMI categories were not substantially altered when the analysis was repeated with additional adjusting for abdominal obesity (Additional file 1: Table S5). Multivariate adjustment for other CVD risk factors such as systolic blood pressure, fasting plasma glucose, triglyceride, high-density lipoprotein cholesterol or total cholesterol decreased the HRs in each obesity category for both men and women but the gender difference remained unchanged (Additional file 1: Table S6). A sensitivity analysis that excluded the first 5 years of follow-up did not alter the results (Additional file 1: Table S7).

## Discussion

Men had a higher CVD mortality than women across all categories of anthropometric measures of obesity after adjusting for age, smoking status, leisure-time physical activity and cohort. The gender difference diminished somewhat in obese individuals especially among diabetic participants.

There is substantial evidence of a gender difference in cardiac autonomic modulation [2-5], lipid and glucose metabolism [6-9], sex hormones [4,10-14] and cytokines [15-19], that might explain part of the observed differences of CVD mortality between women and men. Besides, males tend to have more fat in the abdominal region, even among normal weight or non-obese men, which may be predominantly due to the accumulation of more visceral fat in males than females during the puberty [38].

In addition, sex hormones might play important roles in determining body fat mass and its distribution [11,14], exert multiple direct and indirect effects on insulin and glucose homeostasis or on cardiovascular physiology [4,10,12,13]. Specifically, estrogen increases fat deposition whereas testosterone inhibits fat deposition, and accordingly, men tend to have less overall body fat than women [14], however, the distribution differs between the sexes as discussed below. Women tend to accumulate more subcutaneous fat but less intra-abdominal fat than men probably due to the effects of estrogen by preventing androgen effects [39]. Intra-abdominal fat is believed to be the main pathogenic fat depot that has the clinical relevance to CVD [40], in particular being more metabolically active than adipose depots located in the hip, thigh or buttocks [41]. Clinical studies showed that there was higher intra-abdominal fat accumulation in men than in women for a given level of BMI, WC or WHR [42-44]. In contrast, adult women tend to have a larger hip circumference than men. This is partly due to a difference in bone structure of pelvis, but also in subcutaneous fat mass, that through the metabolically protective physiology of gluteofemoral subcutaneous fat mass, perhaps by trapping excess fatty acids and preventing chronic exposure to elevated lipid levels, or through a beneficial adipokine profile (leptin and adiponectin) [45]. It remains unclear whether CVD risk differs by site of subcutaneous fat accumulation. In addition, estrogen might also play a role in the maintenance of glucose homeostasis and substrate metabolism [46]. Men had significantly higher fasting and significantly lower post-challenge insulin levels than women did [8,47], which is not fully explained by differences in fasting and post-challenge glucose levels between sexes [47]. Additionally, adults have tended to show greater insulin resistance in men than in women [7,9].

Adipose tissue is also a highly active metabolic and endocrine organ, which expresses and secretes a variety of bioactive factors including leptin, adiponectin, and other cytokines [19], which might contribute to the gender difference in CVD mortality. Women have higher circulating leptin levels and higher adiponectin levels than men [15-18]. Hyperleptinemia could be a sign of resistance to normal leptin signaling regulating food intake and satiety. This is believed to be a non-physiological state, which has been associated with an increased risk of diabetes, hypertension and CVD in men but not in women [48,49]. Additionally, hypoadiponectinemia has been found to be associated with an increased risk of both diabetes and CVD [50,51].

Obesity is associated with increased sympathetic activity and decreased vagal activity [52,53], hyperglycemia, insulin resistance [35,54], and is accompanied by chronic low-grade inflammation [19,55], hypertension and dyslipidemia [6,56,57], all of which might predispose to CVD. Abdominal obesity, in particular, is also associated with deficiency of estrogens or testosterone [58,59], although a causal link still needs to be established [14,60]. Deficiency of estrogens or testosterone has been consistently found to be associated with increased risk of CVD [60,61]. Increased leptin and decreased adiponectin levels were observed in obese individuals [48,49,62], but the expression of these cytokines differed between subcutaneous and intra-abdominal fat depots [17,63,64].

Interestingly, the gender difference in CVD mortality appears to somewhat diminish in obese individuals, although misclassification bias between obese and non-obese individuals might occur due to the gender difference of fat distribution, probably enhanced by disturbances of glucose metabolism. Causes of gender difference in CVD mortality with obesity are poorly understood. In our study, the attenuation of the gender difference in CVD mortality among obese individuals remained after adjustment of baseline age or other conventional CVD risk factors, or among non-diabetic individuals even when using other measures of abdominal obesity. The interactions of gender with anthropometric measures were, however, statistically significant only with WC and WHtR in the whole study population and not significant with any of the anthropometric measures in non-diabetic individuals. This suggests an effect modification by diabetes. Studies have indeed shown an attenuation of gender difference in CVD risk once women getting diabetes [24-27,29]. Still, there could be other potential unknown CVD risk factors clustering in obese women due to their older age that contributed to the increased CVD risk in obese women.

Since CVD previously has been considered a ‘male disease’ because of an earlier debut age on the average in men as compared with women, most epidemiological studies have been conducted primarily on men, leading to lesser prevention and treatment efforts in women [65]. Also, obese European women appear to be at greater risk of psychological dysfunction than obese men probably due to increased societal pressures on women to be thin in Europe [66]. In addition, obese women tend to have left ventricular concentric and eccentric hypertrophy, whereas obese men have predominantly concentric hypertrophy, the latter probably being more strongly related to cardiovascular mortality than eccentric hypertrophy, a finding noted recently [30].

Our study was based on several European population-based or occupational studies, with sufficient power to investigate the association between obesity indicators and CVD mortality risk. Yet, limitations of the study exist. The study does not have data on changes in anthropometric measurements before baseline and during follow-up, which makes it impossible to exclude the possibility of ‘reverse causation’ [34], a consequence of underlying diseases before enrolment rather than a cause of deaths. This was examined by excluding the first five years of follow-up, and the results were little affected. Data are based on surveys of Caucasian Europeans, the results may not be applicable to other ethnic groups or races due to difference in percentage of body fat across ethnic groups [67]. Moreover, we do not have data on changes in anthropometric measurements after menopause. Rapid accumulation of abdominal fat after menopause has been suggested to reduce the gender difference in body fat distribution [11,68]. In addition, we do not have information on the use of hormone replacement therapy, which decreases weight gain and the accumulation of intra-abdominal fat in postmenopausal women [69]. Since this is a collaborative data analysis, certain lifestyle variables such as physical activity were recorded differently in different studies. Despite the efforts to “harmonize” variables and to adjust for the different studies in the data analysis, discrepancies exist. The discrepancies in study design and methodologies have been taken into account in our data analysis by fitting “study cohort” into the models as a co-variable, and by making a meta-analysis based on risk estimate of each single study. The effect of each study was also examined by removing each study from the pooled analysis. Exclusion of any study in the analysis had little overall influence on the main findings.

In summary, men had higher CVD mortality than women across all categories of anthropometric measures of obesity, but this gender difference in CVD mortality somewhat diminished in obese individuals, which warrants further investigation to understand the underlying mechanism.

## Appendix

Studies and investigators in this collaborative study are:

**Finland National FINRISK 1987, 1992 and 1997 Cohorts:** J. Tuomilehto^1,2,3^, P. Jousilahti^1^, J. Lindström^1^, 1. Department of Chronic Disease Prevention, National Institute for Health and Welfare, Helsinki; 2. Center for Vascular Prevention, Danube University Krems, Krems, Austria; 3 King Abdulaziz University, Jeddah, Saudi Arabia.

**National FINRISK 2002 Study:** J. Tuomilehto^1,2,3^, T. Laatikainen^1,4,5^, M. Peltonen^1^, J. Lindström^1^, 1. Department of Chronic Disease Prevention, National Institute for Health and Welfare, Helsinki; 2. Center for Vascular Prevention, Danube University Krems, Krems, Austria; 3 King Abdulaziz University, Jeddah, Saudi Arabia; 4. Institute of Public Health and Clinical Nutrition, University of Eastern Finland, Kuopio, Finland; 5. Hospital District of North Karelia, Joensuu, Finland.

**Sweden Northern Sweden MONICA Survey:** S. Söderberg^1,2^, M. Eliasson^1^, 1. Department of Public Health and Clinical Medicine, Umeå University, Umeå, Sweden; 2. Baker IDI Heart and Diabetes Institute, Melbourne, Australia.

**Turkey Turkish Adult Risk Factor Study (TARFS):** A Onat. Department of Cardiology, Cerrahpaşa Medical Faculty, Istanbul University, Istanbul, Turkey.

**United Kingdom Whitehall II Study:** M.G.Marmot^1^, A.G. Tabák^1,2^, M. Kivimäki^1,3^, E.J. Brunner^1^, D.R. Witte^1,4^, 1. Department of Epidemiology and Public Health, University College London, London, UK; 2. Semmelweis University Faculty of Medicine, 1^st^ Department of Medicine, Budapest, Hungary; 3. Finnish Institute of Occupational Health, Helsinki, Finland; 4. Steno Diabetes Center, Gentofte, Denmark.

## Additional file

Additional file 1: Table S1. Baseline characteristics among non-diabetic and diabetic subjects by body mass index and sex. **Table S2.** Baseline characteristics of participants by sex-specific anthropometric measures of abdominal obesity*. **Table S3.** Multivariate-adjusted hazard ratios for cardiovascular disease mortality in men and women by body mass index categories, stratified by cohort*. **Table S4.** Multivariate-adjusted hazard ratios for cardiovascular disease mortality in men and women by body mass index categories: impact of omitting each cohort from the analysis*. **Table S5.** Multivariate-adjusted hazard ratios for cardiovascular disease mortality in men and women by body mass index categories. **Table S6.** Multivariate-adjusted hazard ratios and their 95% confidence intervals for cardiovascular disease (CVD) mortality in men and women by body mass index categories or sex-specific categories of anthropometric measures of abdominal obesity. **Table S7.** Mortality rate per 10 000 person-years and multivariate-adjusted hazard ratios for cardiovascular disease mortality in men and women by body mass index categories or sex-specific categories of anthropometric measures of abdominal obesity, excluding the first five years of follow-up (n= 42 273).
