# Supplementary material for: Obesity attenuates gender differences in cardiovascular mortality
Source: Cardiovasc Diabetol. 2014 Oct 19;13:144. doi: 10.1186/s12933-014-0144-5 (PMC4212094; doi:10.1186/s12933-014-0144-5)
Supplement: Additional file 1: Table S1. — Baseline characteristics among non-diabetic and diabetic subjects by body mass index and sex. Table S2. Baseline characteristics of participants by sex-specific anthropometric measures of abdominal obesity*. Table S3. Multivariate-adjusted hazard ratios for cardiovascular disease mortality in men and women by body mass index categories, stratified by cohort*. Table S4. Multivariate-adjusted hazard ratios for cardiovascular disease mortality in men and women by body mass index categories: impact of omitting each cohort from the analysis*. Table S5. Multivariate-adjusted hazard ratios for cardiovascular disease mortality in men and women by body mass index categories. Table S6. Multivariate-adjusted hazard ratios and their 95% confidence intervals for cardiovascular disease (CVD) mortality in men and women by body mass index categories or sex-specific categories of anthropometric measures of abdominal obesity. Table S7. Mortality rate per 10 000 person-years and multivariate-adjusted hazard ratios for cardiovascular disease mortality in men and women by body mass index categories or sex-specific categories of anthropometric measures of abdominal obesity, excluding the first five years of follow-up (n= 42 273). [file 12933_2014_144_MOESM1_ESM.docx]

**Supplementary Table 1 Baseline characteristics among non-diabetic and diabetic subjects by body mass index and sex**

|  | **<25.0 kg/m^2^** | | |  | **25.0-29.9 kg/m^2^** | | |  | **30.0-34.9 kg/m^2^** | | |  | **≥35.0 kg/m^2^** | | |
| --- | --- | --- | --- | --- | --- | --- | --- | --- | --- | --- | --- | --- | --- | --- | --- |
|  | M | F | P* |  | M | F | P* |  | M | F | P* |  | M | F | P* |
| **Non-diabetic (n=17 056)** | |  |  |  |  |  |  |  |  |  |  |  |  |  |  |
| Age (years) | 49.4 (8.5) | 49.4 (9.3) | 0.67 |  | 51.8 (8.3) | 53.4 (8.2) | 0.00 |  | 53.1 (8.2) | 54.5 (8.4) | 0.00 |  | 53.0 (8.8) | 54.2 (8.8) | 0.14 |
| Abdominal obesity (%)† | |  |  |  |  |  |  |  |  |  |  |  |  |  |  |
| WC | 0.3 | 0.7 | 0.01 |  | 22.6 | 20.2 | 0.02 |  | 92.1 | 72.6 | 0.00 |  | 100.0 | 97.0 | 0.02 |
| WHR | 4.4 | 7.8 | 0.00 |  | 27.0 | 28.5 | 0.17 |  | 65.4 | 47.1 | 0.00 |  | 87.7 | 65.6 | 0.00 |
| WHtR | 0.2 | 0.4 | 0.28 |  | 19.6 | 19.2 | 0.67 |  | 89.7 | 75.8 | 0.00 |  | 100.0 | 98.0 | 0.11 |
| Smoking status (%) | |  | 0.00 |  |  |  | 0.00 |  |  |  | 0.00 |  |  |  | 0.00 |
| Current smokers | 16.9 | 19.5 |  |  | 17.3 | 15.2 |  |  | 18.8 | 13.1 |  |  | 16.6 | 12.9 |  |
| Former smokers | 32.0 | 19.2 |  |  | 38.4 | 18.0 |  |  | 44.0 | 21.1 |  |  | 45.4 | 22.8 |  |
| Physically active (%) | 88.9 | 80.3 | 0.00 |  | 84.5 | 78.7 | 0.00 |  | 77.4 | 70.4 | 0.00 |  | 69.3 | 58.2 | 0.02 |
| SBP (mmHg)‡ | 126 (17) | 124 (20) | 0.00 |  | 134 (18) | 133 (21) | 0.00 |  | 143 (20) | 140 (22) | 0.56 |  | 148 (22) | 145 (24) | 0.23 |
| Serum lipid profiles§ | |  |  |  |  |  |  |  |  |  |  |  |  |  |  |
| TG (mmol/L) | 1.3 (0.9) | 1.0 (0.5) | 0.00 |  | 1.8 (1.2) | 1.4 (0.8) | 0.00 |  | 2.1 (1.4) | 1.6 (0.9) | 0.00 |  | 2.2 (1.2) | 1.7 (0.9) | 0.00 |
| HDL-C (mmol/L) | 1.4 (0.4) | 1.7 (0.4) | 0.00 |  | 1.2 (0.3) | 1.5 (0.4) | 0.00 |  | 1.1 (0.3) | 1.4 (0.4) | 0.00 |  | 1.1 (0.3) | 1.3 (0.4) | 0.00 |
| Total-C (mmol/L) | 6.0 (1.2) | 5.8 (1.2) | 0.00 |  | 6.2 (1.2) | 6.1 (1.3) | 0.00 |  | 6.1 (1.2) | 6.0 (1.3) | 0.09 |  | 5.9 (1.2) | 5.8 (1.2) | 0.58 |
| **Diabetic (n=4499)** | |  |  |  |  |  |  |  |  |  |  |  |  |  |  |
| Age (years) | 52.0 (12.7) | 50.1 (13.4) | 0.01 |  | 55.3 (10.8) | 54.1 (11.7) | 0.02 |  | 57.0 (9.8) | 56.6 (10.7) | 0.52 |  | 56.6 (8.4) | 55.6 (9.8) | 0.32 |
| Abdominal obesity (%)† | |  |  |  |  |  |  |  |  |  |  |  |  |  |  |
| WC | 2.7 | 6.6 | 0.00 |  | 40.3 | 43.4 | 0.21 |  | 91.5 | 84.7 | 0.00 |  | 99.1 | 98.3 | 1.00 |
| WHR | 11.0 | 24.4 | 0.00 |  | 38.4 | 45.7 | 0.00 |  | 68.8 | 60.7 | 0.01 |  | 81.9 | 60.7 | 0.00 |
| WHtR | 3.8 | 11.2 | 0.00 |  | 48.2 | 51.5 | 0.18 |  | 95.9 | 92.4 | 0.03 |  | 99.1 | 99.2 | 1.00 |
| Smoking status (%) | |  | 0.00 |  |  |  | 0.00 |  |  |  | 0.00 |  |  |  | 0.00 |
| Current smokers | 17.8 | 5.8 |  |  | 22.1 | 7.9 |  |  | 21.9 | 7.7 |  |  | 22.4 | 7.8 |  |
| Former smokers | 55.6 | 24.8 |  |  | 45.8 | 20.0 |  |  | 45.5 | 13.7 |  |  | 41.4 | 16.4 |  |
| Physically active (%) | 80.5 | 85.9 | 0.02 |  | 76.3 | 85.4 | 0.00 |  | 71.7 | 77.3 | 0.05 |  | 58.6 | 71.9 | 0.01 |
| SBP (mmHg)‡ | 140 (22) | 136 (21) | 0.22 |  | 149 (20) | 147 (22) | 0.36 |  | 149 (21) | 150 (20) | 0.75 |  | 152 (22) | 150 (22) | 0.44 |
| Serum lipid profiles§ | |  |  |  |  |  |  |  |  |  |  |  |  |  |  |
| TG (mmol/L) | 1.7 (2.0) | 1.3 (0.7) | 0.12 |  | 2.1 (1.6) | 1.6 (1.5) | 0.00 |  | 2.8 (2.1) | 2.2 (1.4) | 0.00 |  | 2.7 (1.6) | 2.6 (1.8) | 0.76 |
| HDL-C (mmol/L) | 1.5 (0.4) | 1.7 (0.5) | 0.00 |  | 1.2 (0.4) | 1.5 (0.4) | 0.00 |  | 1.1 (0.4) | 1.3 (0.4) | 0.00 |  | 1.1 (0.5) | 1.3 (0.5) | 0.00 |
| Total-C (mmol/L) | 5.6 (1.6) | 5.8 (1.5) | 0.33 |  | 5.8 (1.4) | 5.9 (1.3) | 0.11 |  | 5.8 (1.4) | 5.9 (1.4) | 0.71 |  | 5.8 (1.6) | 5.8 (1.5) | 0.69 |

Abbreviations: M, male; F, female; WC, waist circumference; WHR, waist-to-hip ratio; WHtR, waist-to-height ratio; SBP, systolic blood pressure; TG, triglyceride; HDL-C, high-density lipoprotein cholesterol; Total-C, total cholesterol.

Data are means (standard deviations) or as noted.

*Difference between men and women.

†The top quartile of WC, WHR or WHtR in women (90 cm, 0.85 or 0.56) and in men (99 cm, 0.97 or 0.57) was used as the abdominal obesity group.

‡17 050 individuals with available measurement of SBP.

§14 238 individuals with available measurement of TG, 15 632 for HDL-C and 16 983 for Total-C in non-diabetic subjects and 4490 for SBP, 3906 for TG, 4157 for HDL-C and 4435 for Total-C in diabetic subjects, respectively.

**Supplementary Table 2 Baseline characteristics of participants by sex-specific anthropometric measures of abdominal obesity***

|  | **WC <90 (F) / 99 (M)** | | |  | **WC ≥90 (F) / 99 (M)** | | |  | **WHR <0.85 (F) / 0.97 (M)** | | |  | **WHR ≥0.85 (F) / 0.97 (M)** | | |  | **WHtR <0.56 (F) / 0.57 (M)** | | |  | **WHtR ≥0.56 (F) / 0.57 (M)** | | |
| --- | --- | --- | --- | --- | --- | --- | --- | --- | --- | --- | --- | --- | --- | --- | --- | --- | --- | --- | --- | --- | --- | --- | --- |
|  | M (n= 17 337) | F (n= 16 435) | P† |  | M (n= 6298) | F (n= 5524) | P† |  | M (n= 17 759) | F (n= 16 470) | P† |  | M (n= 5876) | F (n= 5489) | P† |  | M (n= 17 740) | F (n= 16 469) | P† |  | M (n= 5895) | F (n= 5490) | P† |
| Age (years) | 46.6 (11.6) | 45.1 (11.9) | 0.00 |  | 51.9 (11.3) | 52.4 (11.7) | 0.02 |  | 46.4 (11.7) | 45.2 (11.9) | 0.00 |  | 52.8 (10.7) | 52.1 (12.0) | 0.00 |  | 46.2 (11.5) | 44.8 (11.8) | 0.00 |  | 53.4 (11.0) | 53.4 (11.5) | 0.82 |
| Diabetes status (%)‡ | | | 0.58 |  |  |  | 0.00 |  |  |  | 0.18 |  |  |  | 0.00 |  |  |  | 0.24 |  |  |  | 0.00 |
| Newly diagnosed diabetes | 11.3 | 11.2 |  |  | 20.2 | 28.8 |  |  | 12.3 | 13.3 |  |  | 17.5 | 24.3 |  |  | 10.0 | 9.7 |  |  | 24.1 | 31.5 |  |
| Known diabetes | 3.8 | 3.5 |  |  | 11.6 | 12.1 |  |  | 3.8 | 3.6 |  |  | 11.8 | 12.2 |  |  | 3.8 | 3.4 |  |  | 11.7 | 12.0 |  |
| Smoking status, % | | | 0.00 |  |  |  | 0.00 |  |  |  | 0.00 |  |  |  | 0.00 |  |  |  | 0.00 |  |  |  | 0.00 |
| Current smokers | 24.5 | 20.2 |  |  | 25.3 | 16.2 |  |  | 23.8 | 19.1 |  |  | 27.3 | 19.5 |  |  | 24.3 | 20.5 |  |  | 25.8 | 15.1 |  |
| Former smokers | 30.3 | 17.6 |  |  | 39.0 | 19.4 |  |  | 30.8 | 17.6 |  |  | 38.2 | 19.5 |  |  | 30.3 | 17.8 |  |  | 39.6 | 18.8 |  |
| Physically active (%) | 82.8 | 78.2 | 0.00 |  | 72.6 | 69.1 | 0.00 |  | 82.5 | 77.8 | 0.00 |  | 72.8 | 70.0 | 0.00 |  | 82.7 | 78.1 | 0.00 |  | 72.1 | 69.3 | 0.00 |
| SBP (mmHg)§ | 130 (18) | 127 (19) | 0.00 |  | 142 (20) | 140 (22) | 0.00 |  | 131 (18) | 128 (20) | 0.00 |  | 141 (20) | 138 (22) | 0.00 |  | 130 (18) | 127 (19) | 0.00 |  | 143 (20) | 141 (22) | 0.00 |
| FPG (mmol/L)§ | 5.4 (1.0) | 5.2 (0.8) | 0.00 |  | 5.9 (1.5) | 5.7 (1.5) | 0.00 |  | 5.4 (1.1) | 5.2 (0.9) | 0.00 |  | 6.0 (1.4) | 5.7 (1.4) | 0.00 |  | 5.4 (0.9) | 5.2 (0.8) | 0.00 |  | 5.9 (1.6) | 5.7 (1.5) | 0.00 |
| TG (mmol/L)§ | 1.5 (1.1) | 1.1 (0.6) | 0.00 |  | 2.2 (1.4) | 1.7 (1.1) | 0.00 |  | 1.5 (1.1) | 1.1 (0.6) | 0.00 |  | 2.1 (1.5) | 1.7 (1.1) | 0.00 |  | 1.5 (1.1) | 1.1 (0.6) | 0.00 |  | 2.2 (1.5) | 1.7 (1.1) | 0.00 |
| HDL-C (mmol/L)§ | 1.3 (0.3) | 1.6 (0.4) | 0.00 |  | 1.1 (0.3) | 1.4 (0.4) | 0.00 |  | 1.3 (0.3) | 1.6 (0.4) | 0.00 |  | 1.1 (0.3) | 1.4 (0.4) | 0.00 |  | 1.3 (0.3) | 1.6 (0.4) | 0.00 |  | 1.1 (0.3) | 1.4 (0.4) | 0.00 |
| Total-C (mmol/L)§ | 5.9 (1.2) | 5.6 (1.2) | 0.00 |  | 6.0 (1.2) | 5.9 (1.2) | 0.37 |  | 5.8 (1.2) | 5.6 (1.2) | 0.00 |  | 6.0 (1.2) | 6.0 (1.3) | 0.29 |  | 5.9 (1.2) | 5.6 (1.2) | 0.00 |  | 5.9 (1.2) | 5.9 (1.2) | 0.92 |

Abbreviations: WC, waist circumference; WHR, waist-to-hip ratio; WHtR, waist-to-height ratio; F, female; M, male.

SBP, systolic blood pressure; FPG, fasting plasma glucose; TG, triglyceride; HDL-C, high-density lipoprotein cholesterol; Total-C, total cholesterol.

Data are means (standard deviations) or as noted.

*The top quartiles of WC, WHR or WHtR in women (90 cm, 0.85 or 0.56) and in men (99 cm, 0.97 or 0.57) were used as the obesity group.

†Difference between men and women.

‡21 555 individuals with information on diabetes status, including 1285 with known diagnosis of diabetes and 20 270 with available measurement of fasting plasma glucose and/or 75 g 2-h oral glucose tolerance test (44.7% women).

§45 573 individuals with available measurement of SBP, 20 759 for FPG, 36 834 for TG, 41 808 for HDL-C and 45 353 for Total-C.

**Supplementary Table 3 Multivariate-adjusted hazard ratios for cardiovascular disease mortality in men and women by body mass index categories, stratified by cohort***

| Study | No. of CVD deaths | BMI <25.0 kg/m^2^ | | |  | BMI 25.0-29.9 kg/m^2^ | | |  | BMI 30.0-34.9 kg/m^2^ | | |  | BMI ≥35.0 kg/m^2^ | | |
| --- | --- | --- | --- | --- | --- | --- | --- | --- | --- | --- | --- | --- | --- | --- | --- | --- |
|  |  | F | M | Sex ratio (M/F) |  | F | M | Sex ratio (M/F) |  | F | M | Sex ratio (M/F) |  | F | M | Sex ratio (M/F) |
| FINRISK (1987) | 412 | 1.0 (Reference) | 2.9 (1.9-4.6) | 2.9 (1.9-4.6) |  | 1.4 (0.9-2.2) | 3.3 (2.2-5.0) | 2.4 (1.7-3.4) |  | 1.3 (0.7-2.2) | 4.8 (3.0-7.5) | 3.8 (2.3-6.3) |  | 3.7 (2.1-6.4) | 6.7 (3.5-12.9) | 1.8 (0.9-3.5) |
| FINRISK (1992) | 207 | 1.0 (Reference) | 2.5 (1.4-4.5) | 2.5 (1.4-4.5) |  | 0.8 (0.4-1.6) | 3.3 (1.9-5.6) | 4.2 (2.3-7.4) |  | 1.5 (0.7-3.1) | 3.0 (1.7-5.6) | 2.1 (1.0-4.1) |  | 2.8 (1.3-5.9) | 8.6 (4.0-18.6) | 3.1 (1.3-7.2) |
| FINRISK (1997) | 266 | 1.0 (Reference) | 2.5 (1.5-4.3) | 2.5 (1.5-4.3) |  | 0.6 (0.3-1.2) | 2.2 (1.3-3.6) | 3.7 (6.4-18.0) |  | 1.5 (0.8-2.9) | 2.4 (1.4-4.2) | 1.6 (0.9-2.7) |  | 1.8 (0.8-3.9) | 3.7 (1.8-7.5) | 2.1 (0.9-4.9) |
| FINRISK (2002) | 83 | 1.0 (Reference) | 3.1 (1.0-9.5) | 3.1 (1.0-9.5) |  | 0.4 (0.1-2.4) | 4.5 (1.6-12.8) | 10.3 (2.4-43.1) |  | 1.7 (0.4-6.3) | 5.4 (1.8-16.4) | 3.3 (1.2-9.0) |  | 2.3 (0.5-10.5) | 4.1 (1.0-16.7) | 1.8 (0.4-8.1) |
| Northern Sweden MONICA (1986) | 67 | 1.0 (Reference) | 3.0 (1.2-7.7) | 3.0 (1.2-7.7) |  | 1.0 (0.3-3.1) | 3.6 (1.5-8.9) | 3.6 (1.4-8.9) |  | 2.0 (0.6-7.3) | 5.3 (1.8-15.3) | 2.6 (0.8-8.7) |  | 4.8 (1.0-23.7) | 5.5 (0.7-46.3) | 1.2 (0.1-12.9) |
| Northern Sweden MONICA (1990) | 42 | 1.0 (Reference) | 2.3 (0.7-7.4) | 2.3 (0.7-7.4) |  | 0.5 (0.1-2.8) | 3.9 (1.4-10.6) | 7.1 (1.6-31.3) |  | 3.5 (0.9-13.0) | 6.1 (1.8-20.2) | 1.7 (0.5-6.2) |  | 2.6 (0.3-22.4) | -- | -- |
| Northern Sweden MONICA (1994) | 53 | 1.0 (Reference) | 3.8 (1.2-11.7) | 3.8 (1.2-11.7) |  | 1.8 (0.5-5.9) | 3.2 (1.1-9.7) | 1.8 (0.8-4.3) |  | 1.3 (0.3-5.8) | 5.4 (1.5-19.3) | 4.2 (1.0-17.2) |  | 1.0 (0.1-9.3) | -- | -- |
| Northern Sweden MONICA (1999) | 17 | 1.0 (Reference) | 3.7 (0.4-34.3) | 3.7 (0.4-34.3) |  | 0.7 (0.0-10.7) | 2.7 (0.3-23.4) | 4.0 (0.5-34.6) |  | -- | 4.3 (0.4-44.3) | -- |  | 3.7 (0.2-67.3) | 9.8 (0.5-177.4) | 2.6 (0.2-43.0) |
| Northern Sweden MONICA (2004) | 0 | -- | -- | -- |  | -- | -- | -- |  | -- | -- | -- |  | -- | -- | -- |
| TARFS (1998-2002) | 90 | 1.0 (Reference) | 1.3 (0.5-3.6) | 1.3 (0.5-3.6) |  | 1.6 (0.6-4.2) | 2.0 (0.8-5.0) | 1.2 (0.6-2.6) |  | 1.3 (0.5-3.8) | 2.6 (0.9-7.2) | 1.9 (0.8-4.8) |  | 2.5 (0.8-7.5) | 1.8 (0.2-15.2) | 0.7 (0.1-5.9) |
| Whitehall II (1991-1993) | 47 | 1.0 (Reference) | 6.0 (1.4-26.1) | 6.0 (1.4-26.1) |  | 0.5 (0.1-5.8) | 6.4 (1.5-28.1) | 12.3 (1.6-94.6) |  | 5.5 (0.9-32.8) | 5.0 (0.7-36.3) | 0.9 (0.2-5.6) |  | -- | 29.4 (2.6-333.2) | -- |
| Pooled | 1284 | 1.0 (Reference) | 2.8 (2.2-3.6) | 2.8 (2.2-3.6) |  | 1.0 (0.8-1.4) | 3.1 (2.5-3.9) | 3.0 (2.4-3.7) |  | 1.6 (1.1-2.1) | 3.8 (2.9-4.9) | 2.4 (1.8-3.2) |  | 2.8 (2.0-3.8) | 5.4 (3.8-7.7) | 2.0 (1.3-2.9) |
| Meta-analysis† | 1284 | 1.0 (Reference) | 3.7 (-0.2-7.7) | 3.4 (0.6-6.2) |  | 0.8 (0.3-1.4) | 3.9 (0.9-6.9) | 6.1 (-2.7-15.0) |  | 1.9 (0.3-3.6) | 4.1 (1.3-6.9) | 2.4 (1.3-3.5) |  | 2.5 (0.5-4.5) | 6.7 (-5.7-19.2) | 1.9 (0.3-3.5) |
| I^2^ (p-value) | -- | -- | 45.3% (0.058) | 17.9% (0.278) |  | 0.0% (0.808) | 26.9% (0.197) | 95.5% (0.000) |  | 0.0% (0.781) | 0.0% (0.471) | 0.0% (45.4) |  | 0.0% (0.951) | 0.0% (0.443) | 0.0%(0.967) |

Abbreviations: CVD, cardiovascular disease; BMI, body mass index; F, female; M, male.

*Hazard ratios (95%CI) from basic Cox proportional hazards model adjusted for baseline smoking status and leisure-time physical activity using attained age as time-scale.

†Meta-analysis estimates were calculated using DerSimonian and Laird random effects models, and statistical heterogeneity was assessed by the I^2^ statistic.

**Supplementary Table 4 Multivariate-adjusted hazard ratios for cardiovascular disease mortality in men and women by body mass index categories: impact of omitting each cohort from the analysis***

|  | BMI <25.0 kg/m^2^ | | |  | BMI 25.0-29.9 kg/m^2^ | | |  | BMI 30.0-34.9 kg/m^2^ | | |  | BMI ≥35.0 kg/m^2^ | | |
| --- | --- | --- | --- | --- | --- | --- | --- | --- | --- | --- | --- | --- | --- | --- | --- |
|  | F | M | Sex ratio (M/F) |  | F | M | Sex ratio (M/F) |  | F | M | Sex ratio (M/F) |  | F | M | Sex ratio (M/F) |
| Omitted study |  |  |  |  |  |  |  |  |  |  |  |  |  |  |  |
| FINRISK (1987) | 1.0 (Reference) | 2.7 (2.1-3.7) | 2.7 (2.1-3.7) |  | 0.9 (0.6-1.3) | 3.1 (2.4-4.1) | 3.5 (2.6-4.6) |  | 1.7 (1.2-2.5) | 3.5 (2.6-4.8) | 2.0 (1.5-2.8) |  | 2.4 (1.6-3.6) | 4.9 (3.2-7.5) | 2.1 (1.3-3.3) |
| FINRISK (1992) | 1.0 (Reference) | 2.8 (2.2-3.7) | 2.8 (2.2-3.7) |  | 1.1 (0.8-1.5) | 3.2 (2.4-4.1) | 2.9 (2.3-3.6) |  | 1.6 (1.1-2.2) | 4.1 (3.1-5.4) | 2.6 (1.9-3.4) |  | 2.8 (1.9-4.0) | 4.9 (3.3-7.3) | 1.7 (1.1-2.7) |
| FINRISK (1997) | 1.0 (Reference) | 2.8 (2.1-3.6) | 2.8 (2.1-3.6) |  | 1.2 (0.9-1.6) | 3.4 (2.6-4.4) | 2.9 (2.3-3.7) |  | 1.6 (1.1-2.2) | 4.3 (3.3-5.7) | 2.8 (2.0-3.7) |  | 3.1 (2.2-4.5) | 5.8 (3.8-8.7) | 1.9 (1.2-2.9) |
| FINRISK (2002) | 1.0 (Reference) | 2.7 (2.1-3.5) | 2.7 (2.1-3.5) |  | 1.1 (0.8-1.4) | 3.1 (2.4-3.9) | 2.9 (2.3-3.6) |  | 1.6 (1.2-2.2) | 3.8 (3.0-5.0) | 2.4 (1.8-3.2) |  | 2.9 (2.1-4.0) | 5.7 (3.9-8.2) | 2.0 (1.3-2.9) |
| Northern Sweden MONICA (1986) | 1.0 (Reference) | 2.7 (2.1-3.5) | 2.7 (2.1-3.5) |  | 1.0 (0.8-1.4) | 3.1 (2.4-3.9) | 3.0 (2.4-3.7) |  | 1.5 (1.1-2.1) | 3.7 (2.9-4.9) | 2.5 (1.9-3.2) |  | 2.7 (1.9-3.7) | 5.2 (3.6-7.4) | 1.9 (1.3-2.9) |
| Northern Sweden MONICA (1990) | 1.0 (Reference) | 2.8 (2.1-3.5) | 2.8 (2.1-3.5) |  | 1.1 (0.8-1.4) | 3.1 (2.5-3.9) | 2.9 (2.4-3.6) |  | 1.5 (1.1-2.1) | 3.8 (2.9-4.9) | 2.5 (1.9-3.2) |  | 2.8 (2.0-3.9) | 5.4 (3.8-7.8) | 2.0 (1.3-2.9) |
| Northern Sweden MONICA (1994) | 1.0 (Reference) | 2.7 (2.1-3.5) | 2.7 (2.1-3.5) |  | 1.0 (0.8-1.4) | 3.1 (2.5-4.0) | 3.1 (2.5-3.9) |  | 1.6 (1.2-2.2) | 3.8 (2.9-4.9) | 2.4 (1.8-3.1) |  | 2.9 (2.1-4.0) | 5.4 (3.8-7.7) | 1.9 (1.3-2.8) |
| Northern Sweden MONICA (1999) | 1.0 (Reference) | 2.7 (2.1-3.5) | 2.7 (2.1-3.5) |  | 1.1 (0.8-1.4) | 3.2 (2.5-4.0) | 3.0 (2.4-3.7) |  | 1.6 (1.2-2.2) | 3.9 (3.0-5.0) | 2.4 (1.9-3.2) |  | 2.8 (2.0-3.9) | 5.3 (3.7-7.6) | 1.9 (1.3-2.8) |
| Northern Sweden MONICA (2004) | 1.0 (Reference) | 2.8 (2.2-3.5) | 2.8 (2.2-3.5) |  | 1.1 (0.8-1.4) | 3.2 (2.5-4.0) | 3.0 (2.4-3.7) |  | 1.6 (1.2-2.1) | 3.9 (3.0-5.0) | 2.5 (1.9-3.2) |  | 2.8 (2.0-3.9) | 5.4 (3.8-7.6) | 1.9 (1.3-2.8) |
| TARFS (1998-2002) | 1.0 (Reference) | 2.9 (2.3-3.8) | 2.9 (2.3-3.8) |  | 1.0 (0.7-1.3) | 3.3 (2.6-4.2) | 3.3 (2.6-4.2) |  | 1.6 (1.2-2.2) | 4.1 (3.1-5.3) | 2.6 (2.0-3.4) |  | 2.8 (2.0-4.0) | 5.7 (4.0-8.2) | 2.0 (1.4-3.0) |
| Whitehall II (1991-1993) | 1.0 (Reference) | 2.7 (2.1-3.5) | 2.7 (2.1-3.5) |  | 1.1 (0.8-1.4) | 3.1 (2.5-3.9) | 2.9 (2.4-3.7) |  | 1.5 (1.1-2.1) | 3.9 (3.0-5.0) | 2.5 (1.9-3.3) |  | 2.8 (2.0-3.9) | 5.2 (3.6-7.5) | 1.8 (1.3-2.7) |
| All studies | 1.0 (Reference) | 2.8 (2.2-3.6) | 2.8 (2.2-3.6) |  | 1.0 (0.8-1.4) | 3.1 (2.5-3.9) | 3.0 (2.4-3.7) |  | 1.6 (1.1-2.1) | 3.8 (2.9-4.9) | 2.4 (1.8-3.2) |  | 2.8 (2.0-3.8) | 5.4 (3.8-7.7) | 2.0 (1.3-2.9) |

Abbreviations: CVD, cardiovascular disease; BMI, body mass index; F, female; M, male.

*Hazard ratios (95%CI) from basic Cox proportional hazards model adjusted for baseline smoking status and leisure-time physical activity using attained age as time-scale.

**Supplementary Table 5 Multivariate-adjusted hazard ratios for cardiovascular disease mortality in men and women by body mass index categories**

|  | BMI, kg/m^2^ | | | |
| --- | --- | --- | --- | --- |
|  | <25.0 | 25.0-29.9 | 30.0-34.9 | ≥35.0 |
| Basic model† | |  |  |  |
| Women | 1.0 (Reference) | 1.0 (0.8-1.4) | 1.6 (1.1-2.1) | 2.8 (2.0-3.8) |
| Men | 2.8 (2.2-3.6) | 3.1 (2.5-3.9) | 3.8 (2.9-4.9) | 5.4 (3.8-7.7) |
| Sex ratio in CVD mortality risk (M/F) | 2.8 (2.2-3.6) | 3.0 (2.4-3.7) | 2.4 (1.9-3.2) | 2.0 (1.3-2.9) |
| Basic model + WC‡ | |  |  |  |
| Women | 1.0 (Reference) | 1.0 (0.7-1.3) | 1.2 (0.9-1.6) | 2.0 (1.4-2.8) |
| Men | 2.8 (2.2-3.6) | 2.8 (2.2-3.5) | 2.8 (2.1-3.7) | 3.9 (2.6-5.7) |
| Sex ratio in CVD mortality risk (M/F) | 2.8 (2.2-3.6) | 2.9 (2.3-3.6) | 2.3 (1.8-3.1) | 2.0 (1.3-2.9) |
| Basic model+WHR‡ |  |  |  |  |
| Women | 1.0 (Reference) | 1.0 (0.7-1.3) | 1.3 (1.0-1.8) | 2.2 (1.6-3.1) |
| Men | 2.9 (2.3-3.7) | 2.9 (2.3-3.6) | 3.0 (2.3-4.0) | 4.1 (2.8-5.9) |
| Sex ratio in CVD mortality risk (M/F) | 2.9 (2.3-3.7) | 3.0 (2.4-3.7) | 2.3 (1.8-3.0) | 1.8 (1.2-2.7) |
| Basic model+WHtR‡ |  |  |  |  |
| Women | 1.0 (Reference) | 0.9 (0.7-1.2) | 1.1 (0.8-1.5) | 1.9 (1.3-2.7) |
| Men | 2.8 (2.2-3.6) | 2.7 (2.1-3.4) | 2.6 (1.9-3.5) | 3.6 (2.5-5.3) |
| Sex ratio in CVD mortality risk (M/F) | 2.8 (2.2-3.6) | 2.9 (2.3-3.6) | 2.4 (1.8-3.1) | 2.0 (1.3-2.9) |

Abbreviations: BMI, body mass index; WC, waist circumference; WHR, waist-to-hip ratio; WHtR, waist-to-height ratio; F, female; M, male; CVD, cardiovascular disease CI, confidence intervals.

†Hazard ratios (95%CI) from basic Cox proportional hazards model adjusted for baseline smoking status, leisure-time physical activity, and cohort using attained age as time-scale.

‡Hazard ratios (95%CI) further adjusting for abdominal obesity, using the top quartile of WC, WHR or WHtR in women (90 cm, 0.85 or 0.56) and in men (99 cm, 0.97 or 0.57) as the abdominal obesity group.

**Supplementary Table 6 Multivariate-adjusted hazard ratios and their 95% confidence intervals for cardiovascular disease (CVD) mortality in men and women by body mass index categories or sex-specific categories of anthropometric measures of abdominal obesity**

|  | BMI, kg/m^2^ | | | |  | WC, cm* | |  | WHR* | |  | WHtR* | |
| --- | --- | --- | --- | --- | --- | --- | --- | --- | --- | --- | --- | --- | --- |
|  | <25.0 | 25.0-29.9 | 30.0-34.9 | ≥35.0 |  | <90 (F) / 99 (M) | ≥90 (F) / 99 (M) |  | <0.85 (F) / 0.97 (M) | ≥0.85 (F) / 0.97 (M) |  | <0.56 (F) / 0.57 (M) | ≥0.56 (F) / 0.57 (M) |
| Basic model (n= 45 594)† | |  |  |  |  |  |  |  |  |  |  |  |  |
| Women | 1.0 (Reference) | 1.0 (0.8-1.4) | 1.6 (1.1-2.1) | 2.8 (2.0-3.8) |  | 1.0 (Reference) | 1.9 (1.6-2.4) |  | 1.0 (Reference) | 1.8 (1.5-2.3) |  | 1.0 (Reference) | 2.1 (1.7-2.6) |
| Men | 2.8 (2.2-3.6) | 3.1 (2.5-3.9) | 3.8 (2.9-4.9) | 5.4 (3.8-7.7) |  | 2.9 (2.4-3.4) | 4.1 (3.4-5.0) |  | 2.7 (2.3-3.2) | 4.2 (3.5-5.1) |  | 3.0 (2.5-3.6) | 4.4 (3.6-5.3) |
| Sex ratio in CVD mortality risk (M/F) | 2.8 (2.2-3.6) | 3.0 (2.4-3.7) | 2.4 (1.9-3.2) | 2.0 (1.3-2.9) |  | 2.9 (2.4-3.4) | 2.1 (1.8-2.6) |  | 2.7 (2.3-3.2) | 2.3 (1.9-2.8) |  | 3.0 (2.5-3.6) | 2.1 (1.8-2.5) |
| Basic model + SBP (n= 45 573)‡ | | |  |  |  |  |  |  |  |  |  |  |  |
| Women | 1.0 (Reference) | 0.9 (0.7-1.3) | 1.3 (1.0-1.8) | 2.2 (1.6-3.1) |  | 1.0 (Reference) | 1.7 (1.4-2.1) |  | 1.0 (Reference) | 1.7 (1.3-2.1) |  | 1.0 (Reference) | 1.8 (1.4-2.2) |
| Men | 2.8 (2.2-3.5) | 2.8 (2.2-3.6) | 3.2 (2.2-4.1) | 4.3 (3.0-6.1) |  | 2.9 (2.4-3.4) | 3.7 (3.1-4.4) |  | 2.7 (2.3-3.2) | 3.9 (3.2-4.6) |  | 2.9 (2.4-3.5) | 3.9 (3.2-4.7) |
| Sex ratio in CVD mortality risk (M/F) | 2.8 (2.2-3.5) | 3.0 (2.4-3.7) | 2.4 (1.9-3.1) | 1.9 (1.3-2.9) |  | 2.9 (2.4-3.4) | 2.2 (1.8-2.6) |  | 2.7 (2.3-3.2) | 2.3 (1.9-2.8) |  | 2.9 (2.4-3.5) | 2.2 (1.8-2.6) |
| Basic model + FPG (n= 20 759)‡ | | |  |  |  |  |  |  |  |  |  |  |  |
| Women | 1.0 (Reference) | 1.2 (0.8-1.7) | 1.6 (1.0-2.4) | 2.8 (1.8-4.4) |  | 1.0 (Reference) | 1.9 (1.4-2.5) |  | 1.0 (Reference) | 2.0 (1.5-2.7) |  | 1.0 (Reference) | 2.1 (1.6-2.8) |
| Men | 2.5 (1.7-3.6) | 3.0 (2.1-4.1) | 3.2 (2.2-4.6) | 3.3 (1.8-5.9) |  | 2.5 (2.0-3.2) | 3.2 (2.5-4.2) |  | 2.4 (1.9-3.0) | 3.5 (2.7-4.5) |  | 2.8 (2.2-3.6) | 3.3 (2.6-4.4) |
| Sex ratio in CVD mortality risk (M/F) | 2.5 (1.7-3.6) | 2.5 (1.9-3.4) | 2.0 (1.4-2.9) | 1.2 (0.7-2.1) |  | 2.5 (2.0-3.2) | 1.7 (1.3-2.2) |  | 2.4 (1.9-3.0) | 1.8 (1.3-2.3) |  | 2.8 (2.2-3.6) | 1.6 (1.2-2.0) |
| Basic model + TG (n= 36 834)‡ | | |  |  |  |  |  |  |  |  |  |  |  |
| Women | 1.0 (Reference) | 0.8 (0.6-1.2) | 1.6 (1.1-2.4) | 2.0 (1.3-3.0) |  | 1.0 (Reference) | 1.8 (1.3-2.3) |  | 1.0 (Reference) | 1.5 (1.2-2.0) |  | 1.0 (Reference) | 1.9 (1.5-2.6) |
| Men | 2.6 (1.9-3.6) | 2.8 (2.1-3.7) | 2.8 (2.0-3.9) | 4.0 (2.6-6.3) |  | 2.7 (2.1-3.4) | 3.8 (2.9-4.8) |  | 2.5 (2.0-3.1) | 3.7 (2.9-4.7) |  | 2.9 (2.2-3.7) | 4.0 (3.1-5.2) |
| Sex ratio in CVD mortality risk (M/F) | 2.6 (1.9-3.6) | 3.3 (2.5-4.4) | 1.8 (1.3-2.4) | 2.1 (1.3-3.4) |  | 2.7 (2.1-3.4) | 2.1 (1.7-2.7) |  | 2.5 (2.0-3.1) | 2.4 (1.9-3.1) |  | 2.9 (2.2-3.7) | 2.1 (1.7-2.6) |
| Basic model + HDL-C (n= 41 808)‡ | | |  |  |  |  |  |  |  |  |  |  |  |
| Women | 1.0 (Reference) | 1.0 (0.7-1.3) | 1.4 (1.0-1.9) | 2.3 (1.7-3.3) |  | 1.0 (Reference) | 1.8 (1.4-2.2) |  | 1.0 (Reference) | 1.7 (1.3-2.1) |  | 1.0 (Reference) | 1.9 (1.5-2.4) |
| Men | 2.4 (1.9-3.1) | 2.5 (1.9-3.1) | 2.9 (2.2-3.8) | 4.0 (2.7-5.7) |  | 2.5 (2.0-3.0) | 3.3 (2.7-4.0) |  | 2.3 (1.9-2.7) | 3.4 (2.8-4.1) |  | 2.5 (2.1-3.1) | 3.5 (2.9-4.3) |
| Sex ratio in CVD mortality risk (M/F) | 2.4 (1.9-3.1) | 2.5 (2.0-3.1) | 2.1 (1.6-2.7) | 1.7 (1.1-2.5) |  | 2.5 (2.0-3.0) | 1.9 (1.5-2.2) |  | 2.3 (1.9-2.7) | 2.0 (1.7-2.5) |  | 2.5 (2.1-3.1) | 1.9 (1.5-2.2) |
| Basic model + Total-C (n= 45 353)‡ | | |  |  |  |  |  |  |  |  |  |  |  |
| Women | 1.0 (Reference) | 1.0 (0.8-1.4) | 1.5 (1.1-2.1) | 2.7 (1.9-3.7) |  | 1.0 (Reference) | 1.9 (1.5-2.4) |  | 1.0 (Reference) | 1.8 (1.5-2.3) |  | 1.0 (Reference) | 2.0 (1.6-2.5) |
| Men | 2.8 (2.2-3.6) | 3.1 (2.5-3.9) | 3.8 (2.9-4.8) | 5.4 (3.8-7.7) |  | 2.9 (2.4-3.5) | 4.1 (3.4-5.0) |  | 2.7 (2.3-3.2) | 4.3 (3.5-5.1) |  | 3.0 (2.5-3.6) | 4.4 (3.6-5.3) |
| Sex ratio in CVD mortality risk (M/F) | 2.8 (2.2-3.6) | 3.1 (2.5-3.8) | 2.5 (1.9-3.2) | 2.0 (1.4-3.0) |  | 2.9 (2.4-3.5) | 2.2 (1.8-2.6) |  | 2.7 (2.3-3.2) | 2.3 (1.9-2.9) |  | 3.0 (2.5-3.6) | 2.1 (1.8-2.6) |
| Basic model + SBP+ FPG (n= 20 744)‡ | | |  |  |  |  |  |  |  |  |  |  |  |
| Women | 1.0 (Reference) | 1.1 (0.7-1.6) | 1.4 (0.9-2.1) | 2.4 (1.5-3.8) |  | 1.0 (Reference) | 1.7 (1.3-2.3) |  | 1.0 (Reference) | 1.8 (1.4-2.5) |  | 1.0 (Reference) | 1.9 (1.4-2.5) |
| Men | 2.5 (1.7-3.6) | 2.8 (2.0-3.9) | 2.8 (1.9-4.0) | 2.8 (1.6-5.1) |  | 2.6 (2.0-3.3) | 3.0 (2.3-3.9) |  | 2.4 (1.9-3.0) | 3.3 (2.6-4.3) |  | 2.8 (2.2-3.6) | 3.1 (2.3-4.0) |
| Sex ratio in CVD mortality risk (M/F) | 2.5 (1.7-3.6) | 2.5 (1.9-3.4) | 2.0 (1.4-2.9) | 1.2 (0.6-2.1) |  | 2.6 (2.0-3.3) | 1.7 (1.3-2.3) |  | 2.4 (1.9-3.0) | 1.8 (1.4-2.4) |  | 2.8 (2.2-3.6) | 1.6 (1.3-2.1) |
| Basic model + SBP+ TG (n= 36 815)‡ | | |  |  |  |  |  |  |  |  |  |  |  |
| Women | 1.0 (Reference) | 0.8 (0.5-1.1) | 1.4 (1.0-2.1) | 1.6 (1.0-2.5) |  | 1.0 (Reference) | 1.6 (1.2-2.1) |  | 1.0 (Reference) | 1.4 (1.1-1.9) |  | 1.0 (Reference) | 1.8 (1.3-2.3) |
| Men | 2.5 (1.9-3.5) | 2.5 (1.9-3.4) | 2.4 (1.8-3.4) | 3.4 (2.2-5.4) |  | 2.7 (2.1-3.4) | 3.4 (2.7-4.4) |  | 2.5 (2.0-3.1) | 3.4 (2.7-4.3) |  | 2.8 (2.2-3.6) | 3.6 (2.8-4.7) |
| Sex ratio in CVD mortality risk (M/F) | 2.5 (1.9-3.5) | 3.3 (2.5-4.4) | 1.7 (1.2-2.4) | 2.1 (1.3-3.5) |  | 2.7 (2.1-3.4) | 2.1 (1.7-2.7) |  | 2.5 (2.0-3.1) | 2.4 (1.9-3.0) |  | 2.8 (2.2-3.6) | 2.1 (1.7-2.6) |
| Basic model + SBP+ HDL-C (n= 41 789)‡ | | | |  |  |  |  |  |  |  |  |  |  |
| Women | 1.0 (Reference) | 0.9 (0.7-1.2) | 1.2 (0.9-1.6) | 1.8 (1.3-2.6) |  | 1.0 (Reference) | 1.6 (1.3-1.9) |  | 1.0 (Reference) | 1.5 (1.2-1.9) |  | 1.0 (Reference) | 1.6 (1.3-2.0) |
| Men | 2.3 (1.8-3.0) | 2.2 (1.7-2.8) | 2.4 (1.8-3.1) | 3.1 (2.1-4.5) |  | 2.4 (2.0-2.9) | 2.9 (2.4-3.5) |  | 2.2 (1.9-2.7) | 3.0 (2.5-3.7) |  | 2.4 (2.0-3.0) | 3.0 (2.5-3.7) |
| Sex ratio in CVD mortality risk (M/F) | 2.3 (1.8-3.0) | 2.5 (2.0-3.1) | 2.0 (1.5-2.6) | 1.7 (1.1-2.5) |  | 2.4 (2.0-2.9) | 1.9 (1.5-2.3) |  | 2.2 (1.9-2.7) | 2.0 (1.7-2.5) |  | 2.4 (2.0-3.0) | 1.9 (1.6-2.3) |
| Basic model + SBP+ Total-C (n= 45 333)‡ | | | |  |  |  |  |  |  |  |  |  |  |
| Women | 1.0 (Reference) | 0.9 (0.7-1.2) | 1.3 (1.0-1.8) | 2.2 (1.6-3.0) |  | 1.0 (Reference) | 1.7 (1.4-2.1) |  | 1.0 (Reference) | 1.7 (1.3-2.1) |  | 1.0 (Reference) | 1.8 (1.4-2.2) |
| Men | 2.7 (2.1-3.5) | 2.8 (2.2-3.5) | 3.2 (2.5-4.1) | 4.3 (3.0-6.2) |  | 2.8 (2.4-3.4) | 3.7 (3.1-4.4) |  | 2.7 (2.3-3.2) | 3.9 (3.2-4.6) |  | 2.9 (2.4-3.5) | 3.9 (3.2-4.7) |
| Sex ratio in CVD mortality risk (M/F) | 2.7 (2.1-3.5) | 3.0 (2.4-3.7) | 2.4 (1.8-3.1) | 2.0 (1.4-2.9) |  | 2.8 (2.4-3.4) | 2.2 (1.8-2.6) |  | 2.7 (2.3-3.2) | 2.3 (1.9-2.8) |  | 2.9 (2.4-3.5) | 2.2 (1.8-2.6) |
| Basic model + FPG+ TG (n= 17 856)‡ | | |  |  |  |  |  |  |  |  |  |  |  |
| Women | 1.0 (Reference) | 1.0 (0.6-1.7) | 1.8 (1.1-3.2) | 2.0 (1.0-3.8) |  | 1.0 (Reference) | 1.9 (1.2-2.9) |  | 1.0 (Reference) | 1.8 (1.2-2.7) |  | 1.0 (Reference) | 2.3 (1.5-3.5) |
| Men | 2.2 (1.3-3.5) | 2.8 (1.8-4.4) | 2.2 (1.3-3.8) | 2.3 (1.0-5.5) |  | 2.4 (1.7-3.5) | 3.2 (2.2-4.6) |  | 2.2 (1.6-3.2) | 3.2 (2.2-4.6) |  | 2.9 (2.0-4.3) | 3.3 (2.2-4.9) |
| Sex ratio in CVD mortality risk (M/F) | 2.2 (1.3-3.5) | 3.0 (1.9-4.5) | 1.2 (0.7-2.0) | 1.2 (0.5-2.9) |  | 2.4 (1.7-3.5) | 1.7 (1.2-2.4) |  | 2.2 (1.6-3.2) | 1.8 (1.3-2.6) |  | 2.9 (2.0-4.3) | 1.5 (1.0-2.0) |
| Basic model + FPG+ HDL-C (n= 19 609)‡ | | | |  |  |  |  |  |  |  |  |  |  |
| Women | 1.0 (Reference) | 1.1 (0.8-1.6) | 1.4 (0.9-2.2) | 2.4 (1.6-3.8) |  | 1.0 (Reference) | 1.7 (1.3-2.3) |  | 1.0 (Reference) | 1.8 (1.3-2.4) |  | 1.0 (Reference) | 1.9 (1.4-2.6) |
| Men | 2.2 (1.5-3.2) | 2.4 (1.7-3.4) | 2.5 (1.7-3.7) | 2.6 (1.4-4.7) |  | 2.2 (1.7-2.8) | 2.6 (2.0-3.5) |  | 2.1 (1.6-2.7) | 2.9 (2.2-3.8) |  | 2.4 (1.8-3.1) | 2.8 (2.1-3.7) |
| Sex ratio in CVD mortality risk (M/F) | 2.1 (1.5-3.2) | 2.2 (1.6-2.9) | 1.8 (1.2-2.6) | 1.1 (0.6-1.9) |  | 2.2 (1.7-2.8) | 1.5 (1.2-2.0) |  | 2.1 (1.6-2.7) | 1.6 (1.2-2.1) |  | 2.4 (1.8-3.1) | 1.4 (1.1-1.8) |
| Basic model + FPG+ Total-C (n= 20 744)‡ | | | |  |  |  |  |  |  |  |  |  |  |
| Women | 1.0 (Reference) | 1.2 (0.8-1.7) | 1.6 (1.0-2.4) | 2.8 (1.8-4.4) |  | 1.0 (Reference) | 1.9 (1.4-2.5) |  | 1.0 (Reference) | 2.0 (1.5-2.6) |  | 1.0 (Reference) | 2.1 (1.6-2.8) |
| Men | 2.5 (1.7-3.6) | 3.0 (2.1-4.2) | 3.2 (2.2-4.6) | 3.3 (1.9-6.0) |  | 2.6 (2.0-3.3) | 3.3 (2.5-4.2) |  | 2.4 (1.9-3.1) | 3.5 (2.7-4.6) |  | 2.8 (2.2-3.7) | 3.4 (2.6-4.4) |
| Sex ratio in CVD mortality risk (M/F) | 2.5 (1.7-3.6) | 2.6 (1.9-3.4) | 2.0 (1.4-3.0) | 1.2 (0.7-2.2) |  | 2.6 (2.0-3.3) | 1.7 (1.3-2.2) |  | 2.4 (1.9-3.1) | 1.8 (1.4-2.4) |  | 2.8 (2.2-3.7) | 1.6 (1.2-2.1) |
| Basic model + TG+ HDL-C (n= 34 349)‡ | | | |  |  |  |  |  |  |  |  |  |  |
| Women | 1.0 (Reference) | 0.8 (0.6-1.2) | 1.6 (1.1-2.3) | 1.8 (1.2-2.8) |  | 1.0 (Reference) | 1.7 (1.3-2.3) |  | 1.0 (Reference) | 1.5 (1.1-2.0) |  | 1.0 (Reference) | 1.9 (1.4-2.6) |
| Men | 2.4 (1.8-3.3) | 2.5 (1.8-3.4) | 2.5 (1.8-3.5) | 3.4 (2.2-5.5) |  | 2.5 (2.0-3.3) | 3.4 (2.6-4.4) |  | 2.3 (1.8-2.9) | 3.3 (2.6-4.3) |  | 2.7 (2.1-3.4) | 3.7 (2.8-4.8) |
| Sex ratio in CVD mortality risk (M/F) | 2.4 (1.8-3.3) | 3.0 (2.2-4.1) | 1.6 (1.2-2.2) | 1.9 (1.2-3.2) |  | 2.5 (2.0-3.3) | 2.0 (1.5-2.5) |  | 2.3 (1.8-2.9) | 2.2 (1.8-2.9) |  | 2.7 (2.1-3.4) | 1.9 (1.5-2.4) |
| Basic model + TG+ Total-C (n= 36 785)‡ | | | |  |  |  |  |  |  |  |  |  |  |
| Women | 1.0 (Reference) | 0.9 (0.6-1.2) | 1.6 (1.1-2.4) | 2.0 (1.3-3.0) |  | 1.0 (Reference) | 1.8 (1.3-2.3) |  | 1.0 (Reference) | 1.5 (1.2-2.0) |  | 1.0 (Reference) | 2.0 (1.5-2.6) |
| Men | 2.6 (1.9-3.5) | 2.8 (2.1-3.7) | 2.8 (2.0-3.9) | 3.9 (2.5-6.2) |  | 2.7 (2.1-3.4) | 3.7 (2.9-4.7) |  | 2.5 (1.9-3.1) | 3.6 (2.8-4.6) |  | 2.8 (2.2-3.6) | 3.9 (3.1-5.1) |
| Sex ratio in CVD mortality risk (M/F) | 2.6 (1.9-3.5) | 3.4 (2.4-4.4) | 1.7 (1.2-2.4) | 2.0 (1.2-3.3) |  | 2.7 (2.1-3.4) | 2.1 (1.7-2.7) |  | 2.5 (1.9-3.1) | 2.4 (1.9-3.0) |  | 2.8 (2.2-3.6) | 2.0 (1.6-2.5) |
| Basic model + SBP+ FPG +TG (n= 17 841)‡ | | | |  |  |  |  |  |  |  |  |  |  |
| Women | 1.0 (Reference) | 0.9 (0.5-1.6) | 1.6 (0.9-2.8) | 1.7 (0.9-3.2) |  | 1.0 (Reference) | 1.7 (1.1-2.6) |  | 1.0 (Reference) | 1.7 (1.1-2.5) |  | 1.0 (Reference) | 2.0 (1.3-3.1) |
| Men | 2.2 (1.3-3.6) | 2.6 (1.7-4.1) | 1.9 (1.1-3.3) | 2.0 (0.8-4.7) |  | 2.4 (1.7-3.5) | 2.9 (2.0-4.2) |  | 2.2 (1.6-3.2) | 3.0 (2.1-4.4) |  | 2.9 (2.0-4.3) | 3.0 (2.0-4.5) |
| Sex ratio in CVD mortality risk (M/F) | 2.2 (1.3-3.6) | 3.0 (1.9-4.5) | 1.2 (0.7-2.0) | 1.2 (0.5-2.9) |  | 2.4 (1.7-3.5) | 1.7 (1.2-2.4) |  | 2.2 (1.6-3.2) | 1.8 (1.3-2.6) |  | 2.9 (2.0-4.3) | 1.5 (1.1-2.1) |
| Basic model + SBP+ FPG +HDL-C (n= 19 594)‡ | | | |  |  |  |  |  |  |  |  |  |  |
| Women | 1.0 (Reference) | 1.0 (0.7-1.5) | 1.2 (0.8-1.9) | 2.1 (1.3-3.2) |  | 1.0 (Reference) | 1.6 (1.2-2.1) |  | 1.0 (Reference) | 1.7 (1.2-2.2) |  | 1.0 (Reference) | 1.7 (1.3-2.3) |
| Men | 2.2 (1.5-3.2) | 2.2 (1.6-3.1) | 2.1 (1.4-3.1) | 2.1 (1.2-3.9) |  | 2.2 (1.7-2.8) | 2.4 (1.8-3.2) |  | 2.1 (1.6-2.7) | 2.7 (2.0-3.6) |  | 2.4 (1.8-3.1) | 2.5 (1.9-3.3) |
| Sex ratio in CVD mortality risk (M/F) | 2.2 (1.5-3.2) | 2.2 (1.6-2.9) | 1.7 (1.2-2.5) | 1.0 (0.6-1.9) |  | 2.2 (1.7-2.8) | 1.5 (1.2-2.0) |  | 2.1 (1.6-2.7) | 1.6 (1.2-2.2) |  | 2.4 (1.8-3.1) | 1.5 (1.1-1.9) |
| Basic model + SBP+ FPG +Total-C (n= 20 729)‡ | | | |  |  |  |  |  |  |  |  |  |  |
| Women | 1.0 (Reference) | 1.1 (0.7-1.6) | 1.4 (0.9-2.1) | 2.4 (1.5-3.8) |  | 1.0 (Reference) | 1.7 (1.3-2.3) |  | 1.0 (Reference) | 1.8 (1.4-2.5) |  | 1.0 (Reference) | 1.9 (1.4-2.5) |
| Men | 2.5 (1.8-3.6) | 2.8 (2.0-3.9) | 2.8 (1.9-4.0) | 2.8 (1.6-5.1) |  | 2.6 (2.0-3.3) | 3.0 (2.3-3.9) |  | 2.4 (1.9-3.1) | 3.3 (2.6-4.3) |  | 2.8 (2.2-3.6) | 3.1 (2.4-4.1) |
| Sex ratio in CVD mortality risk (M/F) | 2.5 (1.8-3.6) | 2.6 (1.9-3.4) | 2.0 (1.4-2.9) | 1.2 (0.7-2.1) |  | 2.6 (2.0-3.3) | 1.7 (1.3-2.3) |  | 2.4 (1.9-3.1) | 1.8 (1.4-2.4) |  | 2.8 (2.2-3.6) | 1.6 (1.3-2.1) |
| Basic model + SBP+ TG +HDL-C (n= 34 330)‡ | | | |  |  |  |  |  |  |  |  |  |  |
| Women | 1.0 (Reference) | 0.8 (0.5-1.1) | 1.3 (0.9-2.0) | 1.4 (0.9-2.3) |  | 1.0 (Reference) | 1.6 (1.2-2.1) |  | 1.0 (Reference) | 1.4 (1.0-1.8) |  | 1.0 (Reference) | 1.7 (1.3-2.3) |
| Men | 2.3 (1.7-3.2) | 2.2 (1.6-3.0) | 2.1 (1.5-2.9) | 2.8 (1.8-4.5) |  | 2.5 (1.9-3.2) | 3.0 (2.3-3.9) |  | 2.2 (1.7-2.8) | 3.0 (2.3-3.9) |  | 2.6 (2.0-3.3) | 3.3 (2.5-4.3) |
| Sex ratio in CVD mortality risk (M/F) | 2.3 (1.7-3.2) | 2.9 (2.2-4.0) | 1.5 (1.1-2.2) | 2.0 (1.2-3.3) |  | 2.5 (1.9-3.2) | 1.9 (1.5-2.5) |  | 2.2 (1.7-2.8) | 2.2 (1.7-2.8) |  | 2.6 (2.0-3.3) | 1.9 (1.5-2.4) |
| Basic model + SBP+ TG +Total-C (n= 36 766)‡ | | | |  |  |  |  |  |  |  |  |  |  |
| Women | 1.0 (Reference) | 0.8 (0.5-1.1) | 1.4 (1.0-2.1) | 1.6 (1.0-2.5) |  | 1.0 (Reference) | 1.6 (1.2-2.1) |  | 1.0 (Reference) | 1.4 (1.1-1.9) |  | 1.0 (Reference) | 1.8 (1.3-2.3) |
| Men | 2.5 (1.8-3.4) | 2.5 (1.9-3.4) | 2.4 (1.7-3.4) | 3.3 (2.1-5.2) |  | 2.6 (2.1-3.3) | 3.3 (2.6-4.2) |  | 2.4 (1.9-3.0) | 3.3 (2.6-4.2) |  | 2.8 (2.1-3.5) | 3.5 (2.7-4.6) |
| Sex ratio in CVD mortality risk (M/F) | 2.5 (1.8-3.4) | 3.2 (2.4-4.3) | 1.7 (1.2-2.3) | 2.1 (1.3-3.4) |  | 2.6 (2.1-3.3) | 2.1 (1.6-2.6) |  | 2.4 (1.9-3.0) | 2.3 (1.8-3.0) |  | 2.8 (2.1-3.5) | 2.0 (1.6-2.5) |
| Basic model + FPG+ TG +HDL-C (n= 16 776)‡ | | | |  |  |  |  |  |  |  |  |  |  |
| Women | 1.0 (Reference) | 0.9 (0.5-1.6) | 1.8 (1.0-3.1) | 1.8 (0.9-3.6) |  | 1.0 (Reference) | 1.8 (1.2-2.8) |  | 1.0 (Reference) | 1.7 (1.1-2.6) |  | 1.0 (Reference) | 2.2 (1.4-3.4) |
| Men | 2.0 (1.2-3.3) | 2.5 (1.6-4.1) | 2,0 (1.1-3.4) | 2.1 (0.9-5.0) |  | 2.3 (1.5-3.3) | 2.9 (1.9-4.3) |  | 2.1 (1.4-3.0) | 2.9 (2.0-4.3) |  | 2.7 (1.8-4.0) | 3.0 (2.0-4.6) |
| Sex ratio in CVD mortality risk (M/F) | 2.0 (1.2-3.3) | 2.7 (1.8-4.2) | 1.1 (0.7-1.9) | 1.1 (0.5-2.8) |  | 2.3 (1.5-3.3) | 1.6 (1.1-2.3) |  | 2.1 (1.4-3.0) | 1.7 (1.2) |  | 2.7 (1.8-4.0) | 1.4 (1.0-2.0) |
| Basic model + FPG+ TG +Total-C (n= 17 854)‡ | | | |  |  |  |  |  |  |  |  |  |  |
| Women | 1.0 (Reference) | 1.0 (0.6-1.7) | 1.8 (1.1-3.2) | 2.0 (1.0-3.8) |  | 1.0 (Reference) | 1.9 (1.2-2.9) |  | 1.0 (Reference) | 1.8 (1.2-2.7) |  | 1.0 (Reference) | 2.3 (1.5-3.5) |
| Men | 2.2 (1.3-3.5) | 2.8 (1.8-4.4) | 2.2 (1.3-3.8) | 2.3 (1.0-5.5) |  | 2.4 (1.7-3.4) | 3.1 (2.1-4.6) |  | 2.2 (1.6-3.2) | 3.2 (2.2-4.6) |  | 2.9 (2.0-4.3) | 3.2 (2.1-4.9) |
| Sex ratio in CVD mortality risk (M/F) | 2.2 (1.3-3.5) | 2.9 (1.9-4.5) | 1.2 (0.7-2.0) | 1.2 (0.5-2.9) |  | 2.4 (1.7-3.4) | 1.7 (1.2-2.4) |  | 2.2 (1.6-3.2) | 1.8 (1.2-2.6) |  | 2.9 (2.0-4.3) | 1.4 (1.0-2.0) |
| Basic model + SBP+ FPG+ TG +HDL-C (n= 16 761)‡ | | | | |  |  |  |  |  |  |  |  |  |
| Women | 1.0 (Reference) | 0.9 (0.5-1.5) | 1.5 (0.9-2.7) | 1.5 (0.8-3.0) |  | 1.0 (Reference) | 1.6 (1.1-2.5) |  | 1.0 (Reference) | 1.6 (1.0-2.4) |  | 1.0 (Reference) | 1.9 (1.2-3.0) |
| Men | 2.0 (1.2-3.3) | 2.3 (1.4-3.7) | 1.7 (0.9-2.9) | 1.7 (0.7-4.0) |  | 2.2 (1.5-3.2) | 2.5 (1.7-3.8) |  | 2.1 (1.4-3.0) | 2.7 (1.8-4.0) |  | 2.6 (1.8-3.9) | 2.6 (1.7-4.0) |
| Sex ratio in CVD mortality risk (M/F) | 2.0 (1.2-3.3) | 2.7 (1.7-4.1) | 1.1 (0.7-1.8) | 1.1 (0.4-2.7) |  | 2.2 (1.5-3.2) | 1.6 (1.1-2.3) |  | 2.1 (1.4-3.0) | 1.7 (1.2-2.4) |  | 2.6 (1.8-3.9) | 1.4 (1.0-2.0) |
| Basic model + SBP+ FPG+ TG +Total-C (n= 17 839)‡ | | | | |  |  |  |  |  |  |  |  |  |
| Women | 1.0 (Reference) | 0.9 (0.5-1.6) | 1.6 (0.9-2.8) | 1.67 (0.9-3.2) |  | 1.0 (Reference) | 1.7 (1.1-2.6) |  | 1.0 (Reference) | 1.7 (1.1-2.5) |  | 1.0 (Reference) | 2.0 (1.3-3.1) |
| Men | 2.2 (1.3-3.6) | 2.6 (1.7-4.1) | 1.9 (1.1-3.3) | 1.9 (0.8-4.6) |  | 2.4 (1.7-3.4) | 2.8 (1.9-4.2) |  | 2.2 (1.5-3.2) | 3.0 (2.0-4.3) |  | 2.9 (1.9-4.2) | 2.9 (1.9-4.4) |
| Sex ratio in CVD mortality risk (M/F) | 2.2 (1.3-3.6) | 2.9 (1.9-4.5) | 1.2 (0.7-2.0) | 1.2 (0.5-2.9) |  | 2.4 (1.7-3.4) | 1.7 (1.2-2.4) |  | 2.2 (1.5-3.2) | 1.8 (1.3-2.6) |  | 2.9 (1.9-4.2) | 1.5 (1.0-2.1) |

Abbreviations: BMI, body mass index; WC, waist circumference; WHR, waist-to-hip ratio; WHtR, waist-to-height ratio; F, female; M, male.

*The top quartiles of WC, WHR or WHtR in women (90 cm, 0.85 or 0.56) and in men (99 cm, 0.97 or 0.57) were used as the obesity group.

†Hazard ratios (95%CI) from basic Cox proportional hazards model adjusting for baseline smoking status, leisure-time physical activity, and cohort using attained age as time-scale.

‡Hazard ratios (95%CI) further adjusting for one or more risk factors, including systolic blood pressure (SBP), fasting plasma glucose (FPG), triglyceride (TG), high-density lipoprotein cholesterol (HDL-C) or total cholesterol (Total-C).

**Supplementary Table 7 Mortality rate per 10 000 person-years and multivariate-adjusted hazard ratios for cardiovascular disease mortality in men and women by body mass index categories or sex-specific categories of anthropometric measures of abdominal obesity, excluding the first five years of follow-up (n= 42 273)**

|  | BMI, kg/m^2^ | | | |  | WC, cm* | |  | WHR* | |  | WHtR* | |
| --- | --- | --- | --- | --- | --- | --- | --- | --- | --- | --- | --- | --- | --- |
|  | <25.0 | 25.0-29.9 | 30.0-34.9 | ≥35.0 |  | <90 (F) / 99 (M) | ≥90 (F) / 99 (M) |  | <0.85 (F) / 0.97 (M) | ≥0.85 (F) / 0.97 (M) |  | <0.56 (F) / 0.57 (M) | ≥0.56 (F) / 0.57 (M) |
| Mortality rate (95%CI) | |  |  |  |  |  |  |  |  |  |  |  |  |
| Women | 10 (8-13) | 19 (15-23) | 31 (24-41) | 58 (42-79) |  | 13 (11-15) | 38 (31-45) |  | 14 (12-16) | 34 (28-42) |  | 12 (10-14) | 42 (35-50) |
| Men | 34 (29-40) | 55 (49-61) | 83 (70-98) | 116 (82-160) |  | 39 (35-43) | 87 (78-98) |  | 40 (36-44) | 95 (84-107) |  | 36 (32-39) | 101 (91-113) |
| Hazard ratios (95%CI)† | |  |  |  |  |  |  |  |  |  |  |  |  |
| Women | 1.0 (Reference) | 1.0 (0.7-1.3) | 1.3 (0.9-1.8) | 2.4 (1.6-3.5) |  | 1.0 (Reference) | 1.8 (1.4-2.3) |  | 1.0 (Reference) | 1.7 (1.3-2.2) |  | 1.0 (Reference) | 1.9 (1.5-2.5) |
| Men | 2.4 (1.9-3.2) | 2.7 (2.1-3.5) | 3.4 (2.5-4.5) | 5.8 (3.9-8.6) |  | 2.6 (2.2-3.2) | 3.8 (3.1-4.7) |  | 2.5 (2.1-3.0) | 4.0 (3.2-4.9) |  | 2.7 (2.2-3.3) | 4.1 (3.3-5.0) |
| Sex ratio in mortality risk (M/F) | 2.4 (1.9-3.2) | 2.8 (2.2-3.5) | 2.6 (1.9-3.6) | 2.4 (1.5-3.7) |  | 2.6 (2.2-3.2) | 2.1 (1.7-2.6) |  | 2.5 (2.1-3.0) | 2.3 (1.8-2.9) |  | 2.7 (2.2-3.3) | 2.1 (1.7-2.6) |

Abbreviations: BMI, body mass index; WC, waist circumference; WHR, waist-to-hip ratio; WHtR, waist-to-height ratio; F, female; M, male; CI, confidence intervals.

*The top quartiles of WC, WHR or WHtR in women (90 cm, 0.85 or 0.56) and in men (99 cm, 0.97 or 0.57) were used as the obesity group.

†Hazard ratios (95%CI) from the Cox proportional hazards model adjusted for baseline smoking status, leisure-time physical activity, and cohort using attained age as time-scale.
